# Supplementary material for: Pull motivation and well-being as drivers of entrepreneurial success: The moderating role of social capital
Source: PLoS One. 2025 Aug 4;20(8):e0327894. doi: 10.1371/journal.pone.0327894 (PMC12321112; doi:10.1371/journal.pone.0327894)
Supplement: S1 Appendix — (DOCX) [file pone.0327894.s001.docx]

Appendix

# Female Entrepreneurial Well-Being Questionnaire

Section I. Mental Health and Well-being

We would like to know your Mental Health and Well-being. Please make the number that corresponds to what you think each question. (1=Never, 2=1-2 times, 3=Once a week, 4= 2 times a week. 5=almost every day, 6=Everyday)

1. How often in the past month did you feel happy?
2. How often in the past month did you feel interested in life?
3. How often in the past month did you feel satisfied with your life?
4. How often during the past month did you feel that you had something important to contribute to the society? (social contribution)
5. How often during the past month did you feel that you belonged to a community (like a social group, your neighborhood, your city, your school)? (social integration)
6. How often during the past month did you feel that our society is becoming a better place for people like you? (social growth)
7. How often during the past month did you feel that people are basically good? (social acceptance)
8. How often during the past month did you feel that the way our society works makes sense to you? (social coherence)
9. How often during the past month did you feel that you liked most parts of your personality? (self-acceptance)
10. How often during the past month did you feel good at managing the responsibilities of your daily life? (environmental mastery)
11. How often during the past month did you feel that you had warm and trusting relationships with others? (positive relationship with others)
12. How often during the past month did you feel that you had experiences that challenged you to grow and become a better person? (personal growth)
13. How often during the past month did you feel confident to think or express your own ideas and opinions? (autonomy)
14. How often during the past month did you feel that your life has a sense of direction or meaning to it? (purpose in life)

Section II Business Performance

This part is concerned with the performance of your company in the past five years. To the best of your knowledge. Please choose the number best estimating how your firm compares to close competitors in your industry on each item.(1= lowest20%, 2= Lower 20%, 3= Middle 20%, 4= Next 20%, 5= Top 20%)

1. After-tax return on total assets.
2. After-tax return on total sales.
3. Firm total sales growth.
4. Overall firm performance and success
5. Your competitive position

Section III. Entrepreneurial Motivation Pull

Please rate your motive for starting your business using the provided numbers for each question from 1 to 7. (1= Absolutely disagree, 7= Absolutely Agree)

1. I would like to make more money

2. I would like to be my own boss.

3. I would like to ensure well-being for myself.

4. I would like to realize myself.

5. I would like to achieve financial independence.

6. I would like to achieve independence in non-financial terms (time)

7. I have a desire for self-development.

8. I would like to be recognized by others.

Section IV. Social Capital

Based on the description, please select the number that best describes your situation. (1= Absolutely disagree, 7= Absolutely Agree)

Bridging cognitive social capital

Acquaintance with entrepreneurial environment

1. You are familiar with public support bodies (such as business angels and government agencies)
2. You have had some special training for young entrepreneurs?
3. You can get some loans under special conditions.
4. You can enter entrepreneurial zones and business incubators.
5. Can you get technical aid for business start-ups?

Bonding cognitive social capital

Closer valuation

1. Many of my immediate family members are engaged in entrepreneurial activities
2. My immediate family values entrepreneurial activity above other activities and careers.
3. Many of my friends are engaged in entrepreneurial activities
4. My friends value entrepreneurial activity above other activities and careers.
5. Many of my colleagues are engaged in entrepreneurial activities
6. My colleagues value entrepreneurial activity above other activities and careers.

Bonding cognitive social capital

Acquaintance with entrepreneurs

1. You know many entrepreneurs in the circle of their family or friends.
2. You have met many entrepreneurs at their workplace or in some other way.
3. You consider entrepreneurs from your circle of family and friends to be “good ones” (1= Not at all, 7= Exetremly good)
4. You consider entrepreneurs met from your workplace to be “good ones”. (1= Not at all, 7= Exetremly good)

Section VI. Profile

1. Gender

A. Male

B. Female

2. Age

A. 18-24

B. 25-34

C. 35-44

D. 45-54

E. 55-64

F. Over 65

3. Education level

A. High school degree and below

B. Some college

C. Bachelor degree

D. Master degree and above

4. Marital status

1. Married
2. Divorce-separated
3. Never married

5. Childbearing status

1. no children
2. Have a child
3. have two children
4. have three or more children

6. role of breadwinner

1. the only breadwinner
2. the main breadwinner (but not the only breadwinner
3. secondary breadwinner
4. No breadwinner responsibilities (or stress)

7. Previous work experience

A. 1-2 years

B. 3-5 years

C. 6-10 years

D.10-20 years

E. More than 20 years

8. Held managerial position

A. Yes

B. No

9. Are you currently involved in entrepreneurial activities?

A. Yes

B. No

10. Entrepreneurial experience

A. Less than 3 years

B. 3-5 years

C. 6-10 years

D. More than 10 years

11.Are you currently working in Micro-business?

A. Yes

B. No

11a. (if Q10 YES) Products or forms currently in operation

1. Maternal and baby products
2. skin care cosmetics
3. agricultural and sideline food
4. clothing shoes bags
5. health products
6. Platform agency retailer (platform product rake sales)
7. other products

11b .(If Q10 YES) Ownership structure

1. Solely owned
2. Team of entrepreneurs
