# Supplementary material for: Pull motivation and well-being as drivers of entrepreneurial success: The moderating role of social capital
Source: PLoS One. 2025 Aug 4;20(8):e0327894. doi: 10.1371/journal.pone.0327894 (PMC12321112; doi:10.1371/journal.pone.0327894)
Supplement: Attachment — Submitted filename: The Effect of.pdf [file pone.0327894.s003.pdf]

# PLOS ONE

# The Effect of Social Capital on the Association Between Mental Health and Well-Being and Business Performance among Female Chinese Entrepreneurs: A Moderated Mediation Model

--Manuscript Draft--

| | |
|---|---|
| **Manuscript Number:** | PONE-D-24-16851 |
| **Article Type:** | Research Article |
| **Full Title:** | The Effect of Social Capital on the Association Between Mental Health and Well-Being and Business Performance among Female Chinese Entrepreneurs: A Moderated Mediation Model |
| **Short Title:** | The Effect of Social Capital on the Association Between Mental Health and Well-Being and Business Performance |
| **Corresponding Author:** | Shibo Li<br>Liaoning University of Technology<br>Jinzhou City, Liaoning Province CHINA |
| **Keywords:** | Female entrepreneurs; Business performance; Mental Health and well-being; Social capital. |
| **Abstract:** | The study examines the mechanism of entrepreneurial motivation pull on business performance. Through the application of regression analysis with SPSS PROCESS macro on a sample of 345 Chinese female entrepreneurs, our findings reveal that mental health and well-being (MWB) plays a fully mediating effect on the promotion of entrepreneurial motivation pull on business performance. Meanwhile, bridge social capital enhances the positive effect of MWB on business performance, while bond social capital buffers this effect. The implications of these findings for research and policy development pertaining to Chinese female entrepreneurs are also discussed. |
| **Order of Authors:** | Shibo Li, PhD student |
| | Edwin SETIAWAN SANUSI, PhD |
| **Additional Information:** | |
| **Question** | **Response** |
| **Financial Disclosure**<br><br>Enter a financial disclosure statement that describes the sources of funding for the work included in this submission. Review the submission guidelines for detailed requirements. View published research articles from *PLOS ONE* for specific examples.<br><br>This statement is required for submission and **will appear in the published article** if the submission is accepted. Please make sure it is accurate. | The author(s) received no specific funding for this work. |

*Powered by Editorial Manager® and ProduXion Manager® from Aries Systems Corporation*

| | |
|---|---|
| **Funded studies**<br>Enter a statement with the following details:<br>• Initials of the authors who received each award<br>• Grant numbers awarded to each author<br>• The full name of each funder<br>• URL of each funder website<br>• Did the sponsors or funders play any role in the study design, data collection and analysis, decision to publish, or preparation of the manuscript?<br><br>Did you receive funding for this work? | |
| **Competing Interests**<br><br>Use the instructions below to enter a competing interest statement for this submission. On behalf of all authors, disclose any competing interests that could be perceived to bias this work—acknowledging all financial support and any other relevant financial or non-financial competing interests.<br><br>This statement is required for submission and **will appear in the published article** if the submission is accepted. Please make sure it is accurate and that any funding sources listed in your Funding Information later in the submission form are also declared in your Financial Disclosure statement.<br><br>View published research articles from *PLOS ONE* for specific examples. | The authors have declared that no competing interests exist. |

*Powered by Editorial Manager® and ProduXion Manager® from Aries Systems Corporation*

| | |
|---|---|
| **NO authors have competing interests**<br><br>Enter: *The authors have declared that no competing interests exist.*<br><br>**Authors with competing interests**<br><br>Enter competing interest details beginning with this statement:<br><br>*I have read the journal's policy and the authors of this manuscript have the following competing interests: [insert competing interests here]*<br><br>* typeset | |
| **Ethics Statement**<br><br>Enter an ethics statement for this submission. This statement is required if the study involved:<br><br>• Human participants<br>• Human specimens or tissue<br>• Vertebrate animals or cephalopods<br>• Vertebrate embryos or tissues<br>• Field research<br><br>Write "N/A" if the submission does not require an ethics statement.<br><br>General guidance is provided below. Consult the submission guidelines for detailed instructions. **Make sure that all information entered here is included in the Methods section of the manuscript.** | Have obtained the IRB exemption letter and uploaded it to the submission system. |

| | |
|---|---|
| **Format for specific study types**<br><br>**Human Subject Research (involving human participants and/or tissue)**<br>• Give the name of the institutional review board or ethics committee that approved the study<br>• Include the approval number and/or a statement indicating approval of this research<br>• Indicate the form of consent obtained (written/oral) or the reason that consent was not obtained (e.g. the data were analyzed anonymously)<br><br>**Animal Research (involving vertebrate animals, embryos or tissues)**<br>• Provide the name of the Institutional Animal Care and Use Committee (IACUC) or other relevant ethics board that reviewed the study protocol, and indicate whether they approved this research or granted a formal waiver of ethical approval<br>• Include an approval number if one was obtained<br>• If the study involved *non-human primates*, add *additional details* about animal welfare and steps taken to ameliorate suffering<br>• If anesthesia, euthanasia, or any kind of animal sacrifice is part of the study, include briefly which substances and/or methods were applied<br><br>**Field Research**<br><br>Include the following details if this study involves the collection of plant, animal, or other materials from a natural setting:<br>• Field permit number<br>• Name of the institution or relevant body that granted permission | |
| **Data Availability**<br><br>Authors are required to make all data underlying the findings described fully available, without restriction, and from the time of publication. PLOS allows rare exceptions to address legal and ethical concerns. See the PLOS Data Policy and FAQ for detailed information. | Yes - all data are fully available without restriction |

*Powered by Editorial Manager® and ProduXion Manager® from Aries Systems Corporation*

| | |
|---|---|
| A Data Availability Statement describing where the data can be found is required at submission. Your answers to this question constitute the Data Availability Statement and **will be published in the article**, if accepted.<br><br>**Important:** Stating 'data available on request from the author' is not sufficient. If your data are only available upon request, select 'No' for the first question and explain your exceptional situation in the text box.<br><br>Do the authors confirm that all data underlying the findings described in their manuscript are fully available without restriction? | |
| **Describe where the data may be found in full sentences. If you are copying our sample text, replace any instances of XXX with the appropriate details.**<br><br>• If the data are **held or will be held in a public repository**, include URLs, accession numbers or DOIs. If this information will only be available after acceptance, indicate this by ticking the box below. For example: *All XXX files are available from the XXX database (accession number(s) XXX, XXX.)*.<br>• If the data are all contained **within the manuscript and/or Supporting Information files**, enter the following: *All relevant data are within the manuscript and its Supporting Information files.*<br>• If neither of these applies but you are able to provide **details of access elsewhere**, with or without limitations, please do so. For example:<br><br>*Data cannot be shared publicly because of [XXX]. Data are available from the XXX Institutional Data Access / Ethics Committee (contact via XXX) for researchers who meet the criteria for access to confidential data.*<br><br>*The data underlying the results presented in the study are available from (include the name of the third party* | All relevant data are within the manuscript and its Supporting Information files. |

| | |
|---|---|
| *and contact information or URL).*<br>• This text is appropriate if the data are owned by a third party and authors do not have permission to share the data.<br><br>* typeset | |
| Additional data availability information: | Tick here if the URLs/accession numbers/DOIs will be available only after acceptance of the manuscript for publication so that we can ensure their inclusion before publication. |

Powered by Editorial Manager® and ProduXion Manager® from Aries Systems Corporation

Manuscript

Click here to access/download;Manuscript;Manuscript_Shibo_LI.docx

# The Effect of Social Capital on the Association Between Mental Health and Well-Being and Business Performance among Female Chinese Entrepreneurs: A Moderated Mediation Model

Shibo Li[1,2¶*], Edwin Setiawan Sanusi[2¶]

[1] Department of Management and Economics, Liaoning University of Technology, Jinzhou, Liaoning Province, China
[2] Department of Management, SolBridge International School of Business, Daejeon, Republic of Korea

[*]Corresponding author

E-mail: sli212@student.solbridge.ac.kr

[¶]These authors contributed equally to this work.

# Abstract

The study examines the mechanism of entrepreneurial motivation pull on business performance. Through the application of regression analysis with SPSS PROCESS macro on a sample of 345 Chinese female entrepreneurs, our findings reveal that mental health and well-being (MWB) plays a fully mediating effect on the promotion of entrepreneurial motivation pull on business performance. Meanwhile, bridge social capital enhances the positive effect of MWB on business performance, while bond social capital buffers this effect. The implications of these findings for research and policy development pertaining to Chinese female entrepreneurs are also discussed.


# Introduction

The number of female entrepreneurs in China is increasing, and their share among entrepreneurs is also gradually rising. In recent years, Chinese women have been actively involved in entrepreneurship, and more and more women are even considering making the transition from start-ups to full-fledged entrepreneurs[1]. But the obstacles encountered by Chinese female entrepreneurs in male-dominated industries reveal the challenges they face in the business environment [2]. And in complex business environments, entrepreneurial motivation is crucial for business success[3].

Based on the Global Entrepreneurship Monitor (GEM) framework, entrepreneurial motivation is divided into entrepreneurial motivation pull and entrepreneurial motivation push [4]. And the entrepreneurial motivation pull emphasizes the

individual's intrinsic interest and passion for entrepreneurial activities [5]. Examining this motivation helps to gain a deeper understanding of the intrinsic motivation of entrepreneurs to start and sustain a business. Although previous studies have explored the relationship between different types of entrepreneurial motivation and different types of business performance[6]. However, it did not delve deeper into the specific mechanisms by which entrepreneurial motivation pull affects business performance.

Moreover, in recent years, there has been a growing interest in the psychological aspects of entrepreneurship [7–9]. Some scholars have also correlated mental health with entrepreneurial motivation as well as firm performance[10]. However, there is a relative paucity of studies that meticulously explore the mechanism of entrepreneurial motivation, especially pull entrepreneurial motivation, on firm performance using mental health and well-being (MWB) as an entry point, especially in the group of Chinese female entrepreneurs.

To address these gaps, this study, from a perspective of MWB, explores the role of MWB in the relationship between entrepreneurial motivation pull and business performance. Specifically, this study will utilize self-determination theory to examine the impact of pull entrepreneurial motivation on business performance. The study proposes that mental health moderates this relationship, while social capital (including bridging and bonding) acts as a moderator, doubly moderating the relationship between mental health and firm performance.

This study is very important and worthwhile. This is because it will help to understand the mechanism of the role of entrepreneurial motivation and firm performance among

Chinese female entrepreneurs. and the important role that mental health and social capital play in it. The study can provide valuable insights for policy makers, practitioners and aspiring entrepreneurs. It can provide a theoretical basis for creating a favorable and positive entrepreneurial environment for women. It also contributes to the existing literature on entrepreneurial motivation, MWB, business performance and social capital.

In the following sections of this paper, we will conduct a detailed literature assessment that led to the formulation of our hypotheses. We will then validate our hypothesis by examining survey data and describing the key findings. Finally, this study explores its contributions, recognizes limits, and proposes options for further research.

# Literature review

## Female Entrepreneurial Motivation Pull

Female entrepreneurial motivation is similar to that of males, and it is described as the process of initiating, guiding, and maintaining goal-oriented actions[11]. It is impacted by a variety of push and pull variables, including cultural settings, educational backgrounds, and individual qualities. Push factors are frequently motivated by need, such as breaking down obstacles like the "glass ceiling" or resolving current issues [12]. Pull factors, on the other hand, are defined as a desire for independence, success, personal growth, and recognition. These entrepreneurs, often known as "opportunity" entrepreneurs, capitalize on developing prospects [13,14]. The Global Entrepreneurship

Monitor (GEM) has been utilizing this categorization method since 2001 [15].

Research has shown that women's entrepreneurial motivation can be a combination of push and pull factors focusing on opportunity, achievement, self-actualization, and helping others[16]. Brahem & Boussema [17]also corroborated the importance of personally relevant (push/pull entrepreneurial motivation) in entrepreneurial endeavors in the field of social media. While focusing on pull entrepreneurial motivation, pull factors represent a completely different form of entrepreneurial motivation from push factors, where women voluntarily choose to start their own business, sometimes even giving up well-paying jobs. Hankinson[18] suggests that the pull factors for women's entrepreneurship include independence, self-actualization, and income-seeking. Jamali[19], on the other hand, by exploring the role of women's human capital in the opportunity identification and utilization, emphasizes the importance of pull factors in opportunity entrepreneurship.

According to the most recent GEM data assessed by Kelley et al.[20]， women are more likely than males to be driven by necessity, with this tendency being particularly noticeable in less developed nations. Female entrepreneurs in less developed countries frequently establish their own firms for survival, nutrition, healthcare, or family-related educational reasons, but in affluent economies, they are unsatisfied with the lack of professional advancement chances[21,22]. The elements that drive entrepreneurship appear to vary according to a country's level of economic development. Female entrepreneurs in less developed nations are more likely to be motivated by necessity, whereas those in affluent economies are more inclined to seek opportunity[23].

## Business Performance in Women-owned businesses

Profits, revenues, return on investment, return on sales, and return on equity are the most often used financial indicators to evaluate firm performance[24]. Objective metrics are used to evaluate organizational performance, which is primarily concerned with monetary gains. Although financial indicators provide a clear and concrete means to assess performance, Dess and Robinson [25] contend that most empirical research on the link between strategic management strategies and organizational performance has conflated "performance" with "success."

The study of the performance of women-owned businesses focuses on its impact factors. Research has shown that resource restrictions, business model pivots, family embeddedness, and demographic variables all have a major influence on the performance of women-owned enterprises[26–28]. Furthermore, research studies have investigated the impact of social capital, financial resources, and household welfare on the performance of women-owned businesses[29–31]. The effect of customer service, credit access programs, and networking on the performance of women-owned enterprises has also been investigated[32–34].

Furthermore, researchers found that female entrepreneurs have specific problems and possibilities which are differ from male entrepreneurs.[35]. Poverty, internet restrictions, and glass ceilings impede their company growth and economic empowerment [36].

## Self-Determination Theory

Self-Determination Theory (SDT) is a macro theory of human motivation, development and wellness[37]. It emphasizes the importance of intrinsic motivation and the fulfillment of psychological needs for autonomy, competence and relatedness [37].

Initially conceived as a social psychological account of the impact of external forces on intrinsic motivation [38], SDT has evolved to encompass a multidimensional conceptualization of motivation, suggesting that different regulations fall along a continuum of self-determination[39]. The theory has been applied in various fields, including health, education and sports. It also highlights the role of autonomy in fostering autonomous behavioral regulation [40].

Moreover, Self-Determination Theory (SDT) is also widely used in the fields of management and entrepreneurship. SDT has been applied to the study of work motivation in the field of management and its relationship to organizational behavior [41]. This concept emphasizes the value of intrinsic motivation and the satisfaction of psychological needs such as relatedness, competence, and autonomy. Experts believe that these elements are essential for the quality of employee motivation [42]. What’s more, studies on the purpose and behavior of entrepreneurs have likewise mostly employed SDT. Alfan and Andriansyah [43] examined the effects of relatedness, autonomy, and competence on university students' propensity for entrepreneurship using SDT. Piperopoulos and Dimov[44] found that when combined with students' self-efficacy beliefs, SDT can either enhance or diminish their intentions for future

entrepreneurial endeavors.

Furthermore, the SDT provides an important theoretical foundation for entrepreneurial studies. The theory of entrepreneurial self-efficacy (ESE) has been developed based on psychological foundations like SDT. Al-Jubari [7] has integrated models of SDT and the Theory of Planned Behavior (TPB) to understand entrepreneurial motivation and intention.

## Mental Health and Well-Being (MWB)

The origins of MWB can be traced back to the broader definition of health put forward by the World Health Organization (WHO) in 1948, which defined health as a state of complete physical, mental, and social adjustment, not merely the absence of disease or infirmity[45]. This basic definition emphasizes that MWB is an integral and important part of overall physical health and emphasizes the holistic nature of well-being.

Scholars believe that MWB consists of four components: subjective experience, positive emotions, psychological functioning, and interpersonal relationships [46]. Positive emotions, autonomy, competence, self-acceptance, personal growth and social connectedness are considered important components of MWB. Also, these elements contribute to an individual's overall well-being, life satisfaction and fulfillment[47].

The concept of MWB is widely used in various fields, such as healthcare and public health. Moreover, in the context of entrepreneurship, MWB plays a crucial role in

shaping entrepreneurial intentions, resilience and success. Research has shown that entrepreneurs with higher levels of MWB are more likely to exhibit higher levels of creativity, problem-solving skills, and adaptability, which are critical to the entrepreneurship and growth of their businesses[48]. Firms can organize to promote innovation, risk-taking and sustainable business practices by fostering entrepreneurial MWB [48].

Additionally, addressing MWB in management and entrepreneurship can improve stress management and interpersonal relationships. By creating a work environment that values mental health, managers can effectively improve employee morale, reduce burnout, and foster a culture of well-being within the organization [49]. Similarly, in entrepreneurship, promoting MWB can help entrepreneurs cope effectively with the uncertainty and stress of running a business, leading to better entrepreneurial outcomes and long-term success[48].

## Social Capital

The concept of social capital, which has garnered substantial attention within academic disciplines, has its origins in the work of influential authors, such as Bourdieu[50], Coleman[51] and Putnam[52]. For Bourdieu[50], social capital is ‘the aggregate of the actual or potential resources which are linked to possession of a durable network of more or less institutionalized relationships of mutual acquaintance and recognition [53].

The definition of social capital has evolved over time, in modern sociology, social

capital is recognized as a valuable resource that influences various outcomes in areas such as health, education, economics, and community development. The concept of social capital has been applied to understand phenomena such as economic performance, environmental collaboration, mental health, and social exclusion, highlighting its versatility and relevance across different contexts [54–56].

Social capital is a multifaceted concept that encompasses various dimensions.

In the dimensions used in this study, bonding social capital and bridging social capital, have been extensively studied by scholars.

Nahapiet & Ghoshal[57] introduced the differentiation between bonding and bridging social capital within the framework of social capital theory. The idea of bridging social capital acutually first came from Granovetter's[58] research. The bridging social capital include weak ties, heterogeneous associational connections, and cross-cutting networks that connect people across different social divisions that would otherwise be isolated from one another, and intimates communities that are socially diverse [59]. In contrast to bonding social capital, bridging social capital is about developing connections with people who belong to different networks. In addition, bridging social capital is associated with benefits such as access to new information, different perspectives, and opportunities for collaboration and innovation [60].

Bonding social capital, a concept rooted in social capital theory, refers to the relationships and connections individuals have within homogeneous groups characterized by similarities in sociodemographic, socioeconomic, or other social

attributes. These connections involve dense ties among individuals who share common characteristics or backgrounds[61].

Studies have highlighted the significance of bonding social capital in various contexts. For instance, bonding social capital has been associated with enhancing collective efficacy within communities[62]. Additionally, bonding social capital has also been linked to well-being in later life [63]. Furthermore, bonding social capital plays a crucial role in disaster preparedness and resilience[64] .

# Hypotheses development

## Entrepreneurial Motivation Pull and MWB

Understanding the motivational drivers behind entrepreneurial endeavors is critical for understanding their impact on entrepreneurs' MWB. Scholars have previously linked the psychological characteristics of entrepreneurs to entrepreneurial characteristics. [65]. Furthermore, entrepreneurial motivation has been linked to happiness and positive psychology, offering a new perspective on how motivation influences entrepreneurs' well-being [66]. When attempting to establish a link between entrepreneurial motivation and happiness, some scholars have demonstrated significant differences in the entrepreneurial well-being achieved by entrepreneurs with various types of motivation [10,67–69]. The MWB differences between opportunity and necessity entrepreneurs were partly explained by the social characteristics of entrepreneurs[67], by education [70], and by entrepreneurial traits, desire for independence and intrinsic

work motivation[71].

For Entrepreneurial Motivation Pull, Spencer and Spencer [72] state that entrepreneurial “motives drive, direct, and select behavior towards certain actions or goals and away from others” could all be considered as pull factors. In the study of Rindova et al.[73], the authors state that the most significant driver of entrepreneurship is the hope to achieve autonomy, or desire for independence. This could be perceived as a pull factor, which is also fundamental for enhancing well-being [10]. Thus, entrepreneurial motivation, particularly the pull factors that attract individuals towards entrepreneurship, has been recognized as a significant contributor to MWB [10].

Furthermore, based on Self-Determination Theory (SDT), Autonomy, competence, and relatedness are considered innate and universal human psychological needs according to Self-Determination Theory (SDT)[74]. Meeting these psychological needs has been associated with increased well-being, job satisfaction, and improved care outcomes for patients [75]. This theory suggests that when entrepreneurs are driven by intrinsic motivations—motivation pull—they experience higher levels of MWB because their work aligns with their personal interests and values, thereby fulfilling their need for autonomy and competence[76,77].

Entrepreneurial motivation pull, characterized by a desire to achieve personal growth, autonomy, and purpose, aligns closely with the principles of SDT[78–80]. When entrepreneurs engage in ventures that are intrinsically motivated, they are more likely to experience a sense of satisfaction and psychological well-being, as their activities are

self-endorsed and aligned with their core values and interests[40]. Building on the literature review, we advance the following hypothese:

Hypothesis 1 (H1): Entrepreneurial Motivation Pull has a positive influence on MWB.

## MWB and Business Performance

MWB significantly impacts the efficiency, creativity, and overall performance of individuals within a business context [81].

Empirical research suggests that employees with higher levels of MWB are more productive, exhibit greater creativity, and have better problem-solving abilities [82]. Moreover, well-being is closely linked to employee engagement; individuals who are mentally healthy are more likely to be committed to their job roles and aligned with their organization’s goals, leading to higher levels of performance and success [83].

Many scholars have successfully argued that mental health significantly affects business performance from different perspectives and in different types of businesses. Jansson & Gunnarsson[84] highlighted the link between MWB and professional capabilities. Kotera et al.[85] examined the relationship between mental health and academic performance, revealing that issues like low self-worth can lead to poor academic outcomes. Hessels et al.[86] discussed the relationship between depression and entrepreneurial exit, demonstrating the importance of mental health on entrepreneurial outcomes. From a small business perspective, Simmons & Dalton[87]

explore how unbalanced job design, occupational uncertainty, and workplace conflict may lead to adverse mental health effects such as depression and anxiety, affecting the well-being of small business owners and, consequently, their business performance. Stephan [10] argued that MWB has a significant positive impact on firm performance from the perspective of the source of entrepreneurial motivation. Therefore, it is proposed that MWB positively influences business performance.

Hypothesis 2 (H2): MWB has a positive influence on Business Performance.

## The central mechanism of MWB

Entrepreneurial motivation is a key driver of business success[88]. The motivation to become an entrepreneur is influenced by various factors, including personal characteristics, social influences, and environmental conditions [89]. Moreover, entrepreneurial motivation has been found to impact small business performance positively [88]. Understanding the determinants of entrepreneurial motivation, such as gender differences, social media entrepreneurship, and cultural contexts, is essential for comprehensively analyzing its effects on business outcomes [17,90]. The mediating role of MWB in this relationship underscores the importance of psychological factors in entrepreneurial success[91].

Entrepreneurs with high levels of MWB are likely to have better decision-making capabilities, motivation, and resource allocation, contributing to improved business performance[87]. MWB enhances an entrepreneur's ability to handle stress, make

strategic decisions, and maintain motivation, thereby facilitating the pursuit of entrepreneurial goals and objectives more effectively [87].

The concept of "motivation pull" refers to the positive attractions or aspirations towards entrepreneurship, such as the desire for autonomy, challenge, and the realization of personal vision [92]. When entrepreneurs have high MWB, they are more resilient to the stresses and challenges of entrepreneurship, which in turn positively affects their motivation and energy to pursue these attractions[93]. This resilience and sustained motivation are crucial for long-term business success and performance [94]. Thus, we propose:

Hypothesis 3 (H3): MWB mediates the relationship between Entrepreneurial Motivation Pull and Business Performance.

## The moderation mechanism of Social Capital

Social capital plays a moderating role in the relationship between MWB and business performance, influencing how individual and collective mental health outcomes can impact organizational success. This mechanism is supported by several studies that have explored it from various perspectives.

From a social capital perspective, social capital, characterized by the networks of relationships among people who live and work in a particular society, can alter the impact of an individual's MWB on their job performance and overall business outcomes

[95]. It does so by providing support, access to resources, and a sense of belonging, which can buffer against the negative effects of poor mental health and enhance the positive effects of good mental health[95].

From a MWB perspective, MWB includes managing stress, regulating emotions, and overall psychological health[96]. These elements are crucial for maintaining productivity and engagement at work [97]. Social capital within the workplace can foster environments where mental health issues are recognized and supported, leading to better employee well-being and organizational performance[98].

Social capital is generally categorized into two primary forms: bridging social capital and bonding social capital [99]. Differences in trust levels between bonding and bridging social capital can influence the relevance of shared information for innovation [40]. Furthermore, distinctions between bonding, bridging, and linking social capital in community initiatives can have varying impacts on innovation and collaboration[100].

Bridging social capital, characterized by inclusive networks that connect individuals across diverse social cleavages, plays a pivotal role in moderating the relationship between MWB and business performance [101]. The essence of bridging social capital lies in its ability to facilitate the flow of information, resources, and support among disparate groups, which is crucial for innovation and adaptability in a business context[102]. Bridging social capital enhances the impact of MWB on business performance by providing individuals access to diverse perspectives and resources, thereby fostering an environment conducive to problem-solving and creativity [102].

Furthermore, bridging social capital contributes to a positive organizational climate by promoting inclusivity and reducing workplace isolation, and this, in turn, supports MWB among employees, creating a virtuous cycle that benefits both individuals and the organization.[103]. It can be inferred that the Bridging Social Capital amplifies the positive impact of MWB on Business Performance

Bonding social capital is a concept that involves close relationships within a group, providing emotional support and a sense of belonging among its members [61]. Bonding social capital, characterized by strong ties within a close-knit group, can foster groupthink, resistance to innovation, and an insular mindset [104]. This type of social capital tends to promote conformity and discourage diverse perspectives, hindering the organization's ability to adapt and grow [104]. Additionally, bonding social capital has been associated with imposing burdens on individuals, limiting their adaptability and potentially leading to detrimental effects on health [105].

It can be seen that high levels of social capital bonding can lead to negative outcomes within the organization, which is detrimental to mental health and in turn affects business performance. This has also been argued by several scholars. Mitchell and Lagory[106] discuss how bonding social capital can increase individuals' levels of mental distress, particularly in impoverished communities. This suggests that close, exclusive networks may not always contribute positively to MWB, potentially impacting business performance. Moreover, the study by Gong et al. [107]delves into the relationship between bonding social capital and psychological well-being among Chinese immigrants in Japan. The research indicates that bonding social capital directly

affects immigrants' psychological well-being, potentially highlighting the adverse effects of strong, insular social connections on mental health, which can subsequently influence business performance. Based on the review of the literature, we propose the following hypotheses.

Hypothesis 4 (H4): Bridging Social Capital enhances the relationship between MWB and Business Performance.

Hypothesis 5 (H5): Bonding Social Capital buffers the relationship between MWB and Business Performance.

In sum, we propose a model in which business non-financial performance plays a critical mediating role (Fig 1).

**Fig1. Theoretical Model.**

# Methodology

## Sample and data collection

This study focuses on the female entrepreneurs in China. The survey was commissioned to be conducted by an online survey platform (www.credemo.com), which targeted a group of respondents whose gender was "'female", whose job status was "entrepreneurial", and whose IP address was in mainland China. The random sampling method was used to obtain the respondent group. The survey conducted for this study was exempted from IRB review by the IRB Board of Directors of SolBridge

International Business School. The decision was based on the fact that the study would not cause any harm to the respondents, other than the fatigue that might result from completing the survey, and that no personally identifiable data was collected. In addition, the key elements and purpose of this questionnaire were briefly summarized in the questionnaire instructions at the outset of the survey, and respondents were given the option to choose whether or not to respond to the questionnaire at their own discretion, and to drop out at any time midway through the process, to ensure that their participation was voluntary.

In February 2024, the survey was conducted through the online survey platform (www.credemo.com) and 405 questionnaires were collected. The questionnaire was answered anonymously. After careful review and elimination of unreasonable answers, 345 valid questionnaires were recovered, accounting for 85.2% of the total. Regression analysis was performed using PROCESS macro of SPSS software.

## Measurement

The questionnaire used in this study is divided into three parts. The first section is about Mental Health and Well-being (MWB). The validated scale, MWB (MHC-SF) scale, which was developed by Keyes[108] was used. A total of 14 items were included to investigate how often the respondent experienced the mental state described in the question in the past month, for example, "How often in the past month did you feel happy?", "How often in the past month did you feel interested in life?" and so on. Responses were on a six-point Likert scale from "never" to "every day".

The second section was on Business Performance. The scale consists of a total of five items to measure the perceived performance of the firm, including "After-tax return on total assets", "After-tax return on total sales", "Firm performance", "After-tax return on total sales", and "After-tax return on total sales". ", "Firm total sales growth", "Overall firm performance and success," "Competitive position" and so on. It was developed from validated research instruments used by Tan and Litsschert [109]. Since the financial performance of most MSMEs is not required to be disclosed, business owners may be hesitant to disclose actual financial data. Therefore, we used a five-point Likert scale from the "lowest 20%" to the "highest 20%" to assess perceived firm performance more accurately. The scale is based on business owners' perceptions of their company's competitive position within their industry over the past five years.

The third section is to measure Entrepreneurial Motivation. A validated Entrepreneurial Motivation scale which was modified and used by Gódány, et al. [110] is used. The pull motivation part of the scale has been selected since we are only studying Entrepreneurial Motivation Pull. The scale contains 9 items, ex, "I would like to make more money", "I would like to be my own boss" and so on. A seven-point Likert scale ranging from "Absolutely disagree" to "Absolutely agree" was adopted to measure the Entrepreneurial Motivation Pull of female entrepreneurs.

The fourth section is about Social Capital. A validated social capital scale which developed by Vuković et al. [111] is used. The scale contains 15 items, which are divided into two dimensions, bridging social capital and bonding social capital. Bridging social capital measures Acquaintance with entrepreneurial environment and

Social valuation. Since the Social valuation is less relevant to our research needs, only the Acquaintance with entrepreneurial environment is retained. Acquaintance with entrepreneurial environment contains 5 items,  ex. "You are familiar with public support bodies (such as business angels and government agencies)"，"You have had some special training for young entrepreneurs?" and so on. Bonding social capital contains 10 items to measure Closer valuation and Acquaintance with entrepreneurs. Bonding social capital takes the form of qualitative and quantitative indicators obtained simultaneously. The item of quantitative indicator is like “Many of my immediate family members are engaged in entrepreneurial activities". The item of qualitative indicator is like "My immediate family values entrepreneurial activity above other activities and careers. " For the statistical results, the corresponding qualitative and quantitative indicators are multiplied to fully measure Bonding social capital. And for the Bridging component, the product is then uniformly divided by 7. Closer valuation contains 6 items, ex "Many of my friends are engaged in entrepreneurial activities "，"My friends value entrepreneurial activity above other activities and careers. "and so on. Acquaintance with entrepreneurs contains 4 items, ex. "You know many entrepreneurs in the circle of their family or friends. "，"You consider entrepreneurs from your circle of family and friends to be ‘good ones’" and so on. A seven-point Likert scale ranging from "Not at all" to "Extremely good" was adopted to measure the social capital of female entrepreneurs.

Previous studies indicated that demographic variables, such as entrepreneur’s education level and entrepreneurial experience, have a significant impact on business

performance [112–115]. Therefore, we controlled for participants' education level and entrepreneurial experience in the following data analysis.

The fifth section of the questionnaire contained 13 different items to gather business profile (microbusiness, products or forms currently in operation and ownership structure) and demographic (gender, age, education level, marital status, childbearing status, role of breadwinner, previous work experience, managerial position and entrepreneurial experience) data.

# Results

## Demographic profile

According to the survey results given in Table 1, the majority of female entrepreneurs surveyed are between the ages of 25 and 44 (77.4%). In terms of education, the majority of respondents had earned a bachelor's degree (64.9 percent). The majority of responders (69.6 percent) were married, and nearly half (49 percent) had only one child. The majority of respondents (42.6 percent) were either the main breadwinners (but not the only one) or secondary breadwinners in their families. Most respondents had prior work experience before beginning their own firm, with the majority of their working years falling between three and ten years (62.1 percent). More than four-fifths (85.5 percent) of respondents have previously held a management role. Nearly nine out of ten respondents (88.4 percent) had less than five years of entrepreneurship experience. Finally, more than two-fifths (41.4 percent) of the female entrepreneurs polled are

involved in micro-business entrepreneurship. Women who start microbusinesses prefer to offer apparel, shoes, and bags (24.5 percent) and skincare cosmetics (28%). The proportion of female micro-business entrepreneurs running single proprietorships (48.3 percent) and team-operated enterprises (51.7 percent) is comparable.

**Table 1. Demographic profiles distribution.**

| Demographic | Value | Frequency | % |
|---|---|---|---|
| Age | 18-24 | 54 | 15.7 |
| | 25-34 | 166 | 48.1 |
| | 35-44 | 101 | 29.3 |
| | 45-54 | 20 | 5.8 |
| | 55-65 | 4 | 1.2 |
| Education | High school degree and below | 20 | 5.8 |
| | Some college | 41 | 11.9 |
| | Bachelor’s degree | 224 | 64.9 |
| | Master’s degree and above | 60 | 17.4 |
| Marital status | Married | 240 | 69.6 |
| | Divorce-separated | 7 | 2.0 |
| | Never married | 98 | 28.4 |
| Childbearing status | No children | 116 | 33.6 |
| | Have a child | 169 | 49.0 |
| | Have two children | 52 | 15.1 |
| | Have three or more children | 8 | 2.3 |
| Role of breadwinner | The only breadwinner | 17 | 4.9 |
| | the main breadwinner (but not the only breadwinner) | 147 | 42.6 |
| | secondary breadwinner | 138 | 40.0 |
| | No breadwinner responsibilities (or stress) | 43 | 12.5 |
| Previous work experience | 1-2 years | 68 | 19.7 |
| | 3-5 years | 103 | 29.9 |
| | 6-10 years | 111 | 32.2 |
| | 10-20 years | 52 | 15.1 |
| | More than 20 years | 11 | 3.2 |
| Ever held managerial position | Yes | 295 | 85.5 |
| | No | 50 | 14.5 |
| Involved in entrepreneurial activities | Yes | 337 | 97.7 |
| | No | 8 | 2.3 |
| Entrepreneurial experience | Less than 3 years | 197 | 57.1 |
| | 3-5 years | 108 | 31.3 |
| | 6-10 years | 32 | 9.3 |
| | More than 10 years | 8 | 2.3 |
| working in Micro-business | Yes | 143 | 41.4 |
| | No | 202 | 58.6 |
| | Maternal and baby products | 16 | 11.2 |

| | | | |
|---|---|---|---|
| Products or forms currently in operation (if Yes for "working in Micro-business") | Skin care cosmetics | 40 | 28.0 |
| | Agricultural and sideline food | 15 | 10.5 |
| | Clothing shoes bags | 35 | 24.5 |
| | Health products | 4 | 2.8 |
| | Platform agency retailer | 21 | 14.7 |
| | Other products | 12 | 8.4 |
| Ownership structure (if Yes for "working in Micro-business") | Solely owned | 69 | 48.3 |
| | Team of entrepreneurs | 74 | 51.7 |

# Descriptive statistics

Inconsistencies in responses in questionnaires can significantly impact the reliability and validity of data collected through surveys [116]. We rescale all the scales in the questionnaire to seven points before data analysis[117].

The descriptive statistics presented in Table 2 reveal that the female entrepreneurs in this study exhibit a higher preference for Social Capital Bridging (M = 5.12, SD = 0.98) compared to Social Capital Bonding (M = 3.57, SD = 1.25). Entrepreneurial motivation Pull (M = 5.91, SD = 0.59) is significantly higher than the average. MWB ((M = 4.68, SD = 1.03 is also slightly above average. Furthermore, the Performance (M = 3.77, SD = 1.16) of the businesses is perceived to be below average.

**Table 2. Descriptive statistics**

| Variables | Obs. | Mean | Std. Dev. | Min | Max |
|---|---|---|---|---|---|
| Pull | 345 | 5.91 | 0.59 | 3.33 | 7.00 |
| MWB | 345 | 4.68 | 1.03 | 1.94 | 6.66 |
| Performance | 345 | 3.77 | 1.16 | 1.00 | 7.00 |
| Bridging | 345 | 5.12 | 0.98 | 1.20 | 7.00 |
| Bonding | 345 | 3.57 | 1.25 | 0.51 | 7.00 |
| Education level | 345 | 2.94 | 0.72 | 1.00 | 4.00 |
| Entrepreneurial experience | 345 | 1.57 | 0.76 | 1.00 | 4.00 |

## Reliability test

The notion of reliably and consistently measuring a phenomenon is referred to as measure reliability, and it is critical in determining its usefulness. In this study, Cronbach's alpha was used to assess the consistency and reliability of the five constructs. The results show that all Cronbach's alpha values are more than 0.72, suggesting strong internal consistency. Furthermore, the aggregate Cronbach's alpha value for all 5 constructs was 0.90, which is much higher than the recommended threshold of 0.7 proposed by Cavana et al. [118], showing that the measures utilized in this study are very reliable.

## Validity test

Construct validity was assessed using factor analysis, which was chosen as the preferred method of measurement. Table 3 shows the factor analysis results. The Kaiser-Meyer-Olkin (KMO) score was 0.864, suggesting adequate sampling coverage. The Bartlett test for sphericity was also statistically significant (p = 0.000; df. = 903), indicating that factor analysis was adequate for the data. These results indicate that the items were unique and supported their respective constructions. In addition, based on the component analysis results, several components with lower coefficients were eliminated.

**Table 3. KMO and Bartlett's test results.**

| | | |
|---|---|---|
| Kaiser-Meyer-Olkin Measure of Sampling Adequacy. | | 0.864 |
| Bartlett's Test of Sphericity | Approx. Chi-Square | 5945.647 |
| | Df. | 903 |

| | | |
|---|---|---|
| | Sig. | .000 |

# Correlation analysis

Table 4 shows the Pearson correlation coefficients for all variables. Entrepreneurial motivation Pull significantly correlates with MWB (r = 0.271, p < 0.01). Research indicates a positive and significant correlation between MWB and company success (r = 0.369, p<0.01). Entrepreneurial motivation Pull correlates positively and significantly with performance (r = 0.164, p < 0.01). Additionally, both moderators, bridging (r = 0.418, p<0.01) and bonding (r = 0.349, p<0.01), have a positive and significant correlation with mental health. Bridging (r = 0.321, p<0.01) and Bonding (r = 0.327, p<0.01) have a positive and significant correlation with performance. Two control variables, educational level (r = 0.139, p<0.01) and entrepreneurial experience (r = 0.254, p<0.01), reveal a positive and significant correlation to business performance.

**Table 4. Correlation matrix.**

| | 1 | 2 | 3 | 4 | 5 | 6 | 7 |
|---|---|---|---|---|---|---|---|
| 1. Pull | 1.000 | | | | | | |
| 2. MWB | 0.271*** | 1.000 | | | | | |
| 3. Performance | 0.164*** | 0.369*** | 1.000 | | | | |
| 4. Bridging | 0.293*** | 0.418*** | 0.321*** | 1.000 | | | |
| 5. Bonding | 0.361*** | 0.349*** | 0.327*** | 0.563*** | 1.000 | | |
| 6. Education level | -0.038 | 0.017 | 0.139*** | 0.161*** | -0.016 | 1.000 | |
| 7. Entrepreneurial experience | 0.149*** | 0.139*** | 0.254*** | 0.022 | 0.153*** | -0.091* | 1.000 |

Note: * *p* <0.1，***p*<0.05, *** *p* <0.01

# Regression analysis

To test the hypothesis, regression analysis making use of PROCESS macro in SPSS,

was done. The lower level and upper level of the regression coefficients were calculated based on 5,000 iterations in a bootstrapping model and 95% level of confidence. The regression outputs were used to test total, direct and indirect effects models [119].

We teste the mediated moderating effect of Bridging Social Capital and Bonding Social Capital on the relationship between MWB and Business Performance, by using Model 16 in the PROCESS macro of SPSS. The Mediated Moderation Model is shown in Fig 2.

**Fig 2. Mediated Moderation Model (Model 16).**

The results of the mediated moderation analysis are shown in Table 5. The control variable, Education level and Entrepreneurial experience are included.

**Table 5. Coefficient of Moderating Mediation effect (Model 16).**

| Variable | β | SE | t | p | LCCI | ULCI |
|---|---|---|---|---|---|---|
| MWB (R-sq=0.0845; p=.0000***) | | | | | | |
| constant | -3.0444 | 0.5937 | -5.1279 | 0.0000*** | -4.2122 | -1.8766 |
| Pull | 0.4513 | 0.0922 | 4.8966 | 0.0000** | 0.2700 | 0.6325 |
| Education level | 0.0521 | 0.0742 | 0.7027 | 0.4827 | -0.0938 | 0.1980 |
| Entrepreneurial experience | 0.1411 | 0.0717 | 1.9693 | 0.0497** | 0.0002 | 0.2821 |
| Performance (R-sq=0.2602; p=.0000***) | | | | | | |
| constant | -0.8535 | 0.6616 | -1.2900 | 0.1979 | -2.1550 | 0.4479 |
| Pull | -0.0409 | 0.1020 | -0.4008 | 0.6888 | -0.2415 | 0.1598 |
| MWB | 0.2977 | 0.0611 | 4.8732 | 0.0000*** | 0.1775 | 0.4179 |
| Bridging | 0.1648 | 0.0743 | 2.2164 | 0.0273** | 0.0185 | 0.3110 |
| Interaction 1 (Bridging X MWB) | 0.1567 | 0.0638 | 2.4577 | 0.0145** | 0.0313 | 0.2822 |
| Bonding | 0.1405 | 0.0560 | 2.5099 | 0.0125** | 0.0304 | 0.2507 |
| Interaction 1 (Bonding X MWB) | -0.1283 | 0.0563 | -2.2776 | 0.0234** | -0.2391 | -0.0175 |
| Education level | 0.2056 | 0.0778 | 2.6437 | 0.0086*** | 0.0526 | 0.3586 |
| Entrepreneurial experience | 0.3076 | 0.0745 | 4.1302 | 0.0000*** | 0.1611 | 0.4540 |

Notes: N=345, β = regression coefficient; SE = standard error of regression coefficient; LLCI=lower limit of the 95% confidence interval; UCLI=upper limit of the 95% confidence interval. * *p* <0.1， ***p*<0.05, *** *p* <0.01

The first hypothesis (H1) assesses whether Entrepreneurial Motivation Pull has a significant impact on the MWB (MWB). According to the findings, Entrepreneurial Motivation Pull significantly affects MWB (ß = 0.4513, SE=0.0922, t = 4.8966, p = 0.0000 < 0.01). Thus, H1 is supported.

The second hypothesis (H2) investigates whether MWB significantly influences the Business Performance. The findings revealed that MWB has a significant effect on Business Performance (ß = 02977, SE=0.0611, t = 4.8732, p = 0.0000 < 0.01). Therefore, H2 is supported.

The third hypothesis (H3) investigates the mediating effect of MWB on the relationship between Entrepreneurial Motivation Pull and Business Performance. The findings reveal a significant indirect effect for the mediation of MWB on the relationship between Entrepreneurial Motivation Pull and Business Performance (indirect effect = .1641, 95% bias-corrected bootstrap CI [0.0821, 0.2614] 95% confidence interval does not include 0). Consequently, H3 is supported. Moreover, the mediation is complete indirect effect of Entrepreneurial Motivation Pull on business performance.

**Table 6. Bootstrap results for moderated mediation effect.**

| Moderator | Effect | SE | LCCI | ULCI |
|---|---|---|---|---|
| Bridging | 0.0707 | 0.0364 | 0.0042 | 0.1467 |
| Bonding | -0.0579 | 0.0304 | -0.1233 | -0.0053 |

Notes: SE = standard error of regression coefficient; LLCI=lower limit of the 95% confidence interval; UCLI=upper limit of the 95% confidence interval.

To estimate the hypothesized moderated mediation effects, we used a parametric bootstrap procedure. With 5,000 Monte Carlo replications, the results showed in table 6.

**Fig 3. Moderating effects of Bridging Social Capital and Bonding Social Capital on the relationship between MWB and Business Performance.**

The association between relationship between MWB and Business Performance was plotted with three fit lines when the levels of Bonding Social Capital were 1SD below, 0 and 1SD above the mean. This represents the simple moderating effect of Bonding Social Capital at three levels. At the same time, the three charts of the relationship between MWB and Business Performance moderated by Bridging Social Capital are plotted at 1SD, 0 and 1SD above the mean respectively. To reflect the moderating effects of Bridging Social Capital and Bonding Social Capital on the relationship between MWB and Business Performance.

In the Fig 3, the slope of the high Bonding Social Capital's fit line is always lower than that of the low Bonding Social Capital's fit line. As the bonding value increases (e.g., .00 to .99), the slope becomes flatter. This suggests that the effect of MWB on Business Performance diminishes at higher bonding. Additionally, in conjunction with the location of the intersection of the three Bonding Social Capital's fit lines at the same level of Bridging Social Capital, it can be inferred that the Bonding Social Capital has an antagonistic effect as a moderating variable. That is, the moderating effect of Bonding Social Capital is opposite to the original causal relationship between MWB and Business Performance. And it reduces the strength of the relationship between MWB and Business Performance. What's more, comparing the three charts in Fig 3, the slopes of the fit lines decrease as the value of Bridging Social Capital decreases, when the level of Bonding Social Capital is kept constant. It reveals that the moderator

variable Bridging Social Capital has a synergistic effect. That is, the moderator variable Bridging Social Capital strengthens the positive relationship between MWB and Business Performance.

The fourth hypothesis (H4) explores whether Bridging Social Capital positively moderates the relationship between MWB and Business Performance. The results (Table 6, Fig 3) indicate that Bridging Social Capital does indeed have a significant positive moderating effect on the relationship between MWB and Business Performance (moderated mediation index= 0.0707, SE =0.0364, bias-corrected bootstrap CI [0.0042, 0.1467] 95% confidence interval does not include 0). As a result, H4 is supported.

The fifth hypothesis (H5) investigates whether Bonding Social Capital negatively moderates the relationship between MWB and Business Performance. The results (Table 6, Fig 3) reveal that Bonding Social Capital does indeed have a significant negative moderating effect on the relationship between MWB and Business Performance (moderated mediation index=-0.0579, SE=0.0304, bias-corrected bootstrap CI [-0.1233,-0.0053] 95% confidence interval does not include 0). H5 is supported.

**Table 7. Summary of results for the research hypotheses.**

| Hypothesis | | Empirical Validation |
|---|---|---|
| H1 | Entrepreneurial Motivation Pull has a positive influence on MWB. | Supported |
| H2 | MWB has a positive influence on Business Performance. | Supported |
| H3 | MWB mediates the relationship between Entrepreneurial Motivation Pull and Business Performance. | Supported |
| H4 | Bridging Social Capital enhances the relationship between MWB and Business Performance. | Supported |

| | | |
|---|---|---|
| H5 | Bonding Social Capital buffers the relationship between MWB and Business Performance. | Supported |

The summary of results for hypothesis testing are presented in Table 7.

# Discussion

This study meticulously examines the complex interplay between entrepreneurial motivation, MWB, and business performance in Chinese female entrepreneurs, underscoring the critical moderating role of social capital[114]. The findings indicate that entrepreneurial motivation positively affects MWB. Consequently, enhanced MWB significantly boosts business performance. MWB fully mediates the relationship between entrepreneurial motivation pull and business performance. Moreover, social capital plays a crucial moderating role in the mental wellbeing-business performance nexus. In this context, bridging social capital enhances, while bonding social capital diminishes, the positive impact of mental health on company performance.

Initially, the study examines the interactions between entrepreneurial motivation, MWB, and business performance. The study supports Stephan's [10] findings by stressing the favorable influence of entrepreneurial motivational pull on MWB. This emphasizes the importance of intrinsic motivators in enhancing entrepreneurial well-being. Furthermore, the findings highlight the importance of mental health in driving entrepreneurial success, which is similar with the findings of Hessels et al. [120].

The finding that MWB mediates entrepreneurial motivational pull and business performance is also consistent with theoretical analysis[87] .

The complete mediating role of MWB in the findings then highlights the importance of addressing entrepreneurial mental health as a strategy to promote business success. It is clear that companies may prioritize mental health as a key strategic approach to promoting entrepreneurial business success.

Addressing MWB enhances entrepreneurs' psychological state and is pivotal in optimizing performance and productivity. This mediation impact underlines the link between mental health, entrepreneurial motivation, and business effectiveness. It also underlines the necessity for comprehensive support structures within the entrepreneurial ecosystem.

Additionally, the research explores social capital's influence on the mental health-commercial performance relationship. Bridging social capital strengthens the positive influence of mental health on business performance by connecting different social groups and facilitating the sharing of information and resources. This finding is compatible with the work of Pihl et al.[103]. This emphasizes the importance of a broad social network for entrepreneurial success.

On the contrary, the negative buffering effect of bonding social capital is consistent with Gong et al.'s[107] findings. It can be seen that while tight networks provide support and solidarity, they may limit the flow of new ideas and information, potentially hindering the innovation and adaptability of entrepreneurial firms.

The present study identifies these contrasting effects, emphasizing the need to balance the benefits of close relationships with the advantages of diverse connections in entrepreneurial environments.

Overall, this research underscores the critical impact of social capital dynamics on business performance. The study also emphasizes the differences in the effects of bridging and bonding social capital on the relationship between mental health and company performance. Firms can improve access to new resources and views by using bridging social capital, but understanding the limitations of bonding social capital is critical for boosting entrepreneurial innovation and adaptation. These findings suggest a more sophisticated picture of the entrepreneurial environment, in which psychological and social aspects play a crucial role. Encouraging a supportive entrepreneurial environment that promotes psychological well-being and leverages varied social networks may be critical to maximizing entrepreneurs' potential and boosting entrepreneurship.

# Conclusion and Implications

The study offers a nuanced view of how entrepreneurial motivation, MWB, and social capital interact to affect business performance. It confirms the hypothesized relationships, showing that MWB is a critical mediator between motivation and performance and a key indicator of entrepreneurial health and success.

The study also reveals the dual nature of social capital: while bridging capital enhances performance through inclusive networks, bonding capital, despite its support, may restrict growth due to limited perspectives. The findings underscore the need for an environment that leverages MWB and social capital to maximize entrepreneurial potential.

Theoretically, the study contributes to the literature by highlighting the importance of intrinsic motivation and MWB in business performance and by examining the moderating role of social capital, offering a more holistic framework for entrepreneurial research.

Practically, it suggests that Chinese women entrepreneurs can improve business performance by fostering MWB and creating a supportive network. Policies should address mental health and social capital alongside financial support, with a focus on external connections to avoid the negative impacts of insular networks.

# Limitation and future research direction

Firstly, the main population studied in this research is Chinese women entrepreneurs. This may limit the generalization of the findings to other populations and geographies. This is because aspects of China-specific family views (e.g., men dominate outside the home, women dominate inside the home), social perceptions (e.g., men are superior to women in some areas of China), and economic contexts may have an impact on the relationship between entrepreneurial motivation, psychological well-being, and business performance among Chinese women entrepreneurs. And this effect may not be applicable in other countries and regions or other populations. Thus, in future research, it is possible to explore how cultural and socio-economic factors influence the various factors of the model, especially considering whether social capital operates in a consistent manner across different social contexts.

Secondly, the study relied on questionnaires to obtain respondents' own predicted indicators of MWB, social capital and perceived business performance. However, these results may introduce bias. For example, respondents may have overestimated or underestimated their well-being or business performance. Therefore, in future studies, attempts could be made to obtain objectively measured data (e.g., externally disclosed business performance data) to supplement respondents' own predictions. This will effectively improve the accuracy of the study and the rigor of the results.

In addition, this study utilizes questionnaire research. The cross-sectional data obtained in this way limits the ability to infer causal relationships. If a longitudinal study could have been incorporated, it would have provided a deeper understanding of how entrepreneurial motivation, MWB, and social capital dynamically affect business performance over time. In the future, longitudinal studies could be included, which could provide insight into how these relationships evolve over time, particularly the importance of social capital and MWB on business performance in response to major life or global events.

# References


1. Van der Zwan P, Verheul I, Thurik AR. The entrepreneurial ladder, gender, and regional development. Small Business Economics. 2012 Sep;39(3):627–43.
2. Alhajri A, Aloud M. Female digital entrepreneurship: a structured literature review. International Journal of Entrepreneurial Behavior & Research. 2024 Mar 11;30(2/3):369–97.
3. Powell BC, Donohue JM, Liang X, Fox JB. Sales growth of Chinese private owned enterprises (POEs): an exploratory analysis. Journal of Chinese Entrepreneurship. 2013 May 30;5(2):104–21.

4. Alam MS, Biswas K, Sulphey MM. A Case Study on the Entrepreneurial Process of Push and Pull Women Entrepreneurs. South Asian Journal of Business and Management Cases. 2021 Aug 4;10(2):207–17.

5. Wing CK. Understanding the Pull Motivations of Malaysian Women Music Teachers as Music Entrepreneurs. Vol. 7, Malaysian Journal of Music. 2018.

6. Li S, Sanusi ES. Entrepreneurial motivations and business performance: A study of female online microbusiness owners. PLoS One. 2023 Aug 1;18(8 August).

7. Al-Jubari I. College students' entrepreneurial intention: Testing an integrated model of SDT and TPB. Sage Open. 2019 Apr 1;9(2):1–15.

8. Margaça C, Hernández-Sánchez B, Sánchez-García JC, Cardella GM. The Roles of Psychological Capital and Gender in University Students' Entrepreneurial Intentions. Front Psychol. 2021 Jan 15;11.

9. Hu W, Xu Y, Zhao F, Chen Y. Entrepreneurial Passion and Entrepreneurial Success—The Role of Psychological Capital and Entrepreneurial Policy Support. Front Psychol. 2022 Feb 25;13.

10. Stephan U. Entrepreneurs' Mental Health and Well-Being: A Review and Research Agenda Journal: Academy of Management Perspectives Academy of Management Perspectives ENTREPRENEURS' MENTAL HEALTH AND WELL-BEING: A REVIEW AND RESEARCH AGENDA. Academy of Management Perspectives. 2018;3(32):290–322.

11. Goyal PK. Motivation: Concept, theories and practical implications. [Internet]. Available from: http://www.casirj.com

12. Huang X. Influence Factors and Motivation of Women Entrepreneurs in Greater Bay Area. In 2022. p. 1424–37.

13. Block JH. OPPORTUNITY RECOGNITION AND EXPLOITATION BY NECESSITY AND OP-PORTUNITY ENTREPRENEURS: EMPIRICAL EVIDENCE FROM EARNINGS EQUATIONS.

14. Reynolds P, Bosma N, Autio E, Hunt S, De Bono N, Servais I, et al. Global entrepreneurship monitor: Data collection design and implementation 1998-2003. Small Business Economics. 2005 Apr;24(3):205–31.

15. Raynolds PD, Michael S. Camp, William D. Bygrave, Erkko Autio, Michael Hay. Global entrepreneurship monitor: 2001 executive report. 2001.

16. Almobaireek WN, Manolova TS. Entrepreneurial motivations among female university

youth in Saudi Arabia. Journal of Business Economics and Management. 2013;14(SUPPL1).

17. Brahem M, Boussema S. Social media entrepreneurship as an opportunity for women: The case of Facebook-commerce. The International Journal of Entrepreneurship and Innovation. 2023 Aug 10;24(3):191–201.

18. Hankinson A. The key factors in the profiles of small firm owner-managers that influence business performance. The South Coast Small Firms Survey, 1997-2000. Industrial and Commercial Training. 2000 Jun 1;32(3):94–8.

19. Jamali D. Constraints and opportunities facing women entrepreneurs in developing countries: A relational perspective. Gender in Management: An International Journal. 2009 Jun 12;24(4):232–51.

20. Kelley DJ, Singer S, Herrington MD. Global entrepreneurship monitor 2011 global report 2011. 2012.

21. Belcourt M. A Family Portrait of Canada’s Most Successful Female Entrepreneurs The road to self-employment. 1990.

22. Holly Buttner E. Women’s organizational exodus to entrepreneurship: Self-reported motivations and correlates with success [Internet]. 1997. Available from: https://www.researchgate.net/publication/279548467

23. van der Zwan P, Thurik R, Verheul I, Hessels J. Factors influencing the entrepreneurial engagement of opportunity and necessity entrepreneurs. Eurasian Business Review. 2016 Dec 3;6(3):273–95.

24. Kaplan RS, Norton DP. The Balanced Scorecard: Measures that Drive Performance. Harv Bus Rev. 1992;1(70):71–9.

25. Dess GG, Robinson RB. Measuring Organizational Performance in the Absence of Objective Measures: The Case of the Privately-Held Firm and Conglomerate Business Unit [Internet]. Vol. 5, Source: Strategic Management Journal. Available from: https://www.jstor.org/stable/2486280?seq=1&cid=pdf-reference#references_tab_contents

26. Chavan，Meena. Framework Identifying the Heterogeneous Resource Constraints Faced by Women Entrepreneurs at the Planning and Managing Stages. Vol. 6, International Journal of Interdisciplinary Social Sciences. 2011. p. 1–22.

27. Mari M, Poggesi S, De Vita L. Family embeddedness and business performance: evidences from women-owned firms. Management Decision. 2016 Mar 21;54(2):476–

500.

28. Vasan M. MODERATING EFFECT OF DEMOGRAPHIC AND BUSINESS CHARACTERISTICS ON PERFORMANCE OF WOMEN OWNED SMALL ENTERPRISES: EMPIRICAL EVIDENCE FROM INDIA. Serbian Journal of Management. 2020;15(2):319–33.

29. Tundui CS, Tundui H. An Empirical Analysis of Social Capital and Enterprise Performance in Tanzania: The Case of Women Owned Businesses. International Journal of Developing Societies. 2013 Mar 27;2(1).

30. Adam A, Wan Abdullah WR, Maruhun ENS, Anwar ISK, Salin ASAP. The Resource-Based View Theory and Women Microbusiness Entrepreneurs: A Contribution to Business Sustainability. International Journal of Academic Research in Business and Social Sciences. 2022 Oct 11;12(10).

31. Seshie-Nasser HA, Oduro AD. Women-owned businesses and household welfare. International Journal of Gender and Entrepreneurship. 2018 Nov 8;10(4):310–31.

32. Coleman S, Carsky M. Women Owned Businesses and Bank Switching: The Role of Customer Service. The Journal of Entrepreneurial Finance. 1996 Dec 1;5(1):75–83.

33. Waweru CW, Wangui P, Walter N, Bichanga O. Role of Credit Access Program on the Performance of Women Owned MSES Incubated at KIRDI. Journal of Entrepreneurship &Amp [Internet]. 2022;1(6):1–20. Available from: https://doi.org/10.53819/81018102t4029

34. Miller NJ, Besser TL, Vigna D. Networking among apparel store owners in small U.S. communities: Developing social capital. Clothing and Textiles Research Journal. 2011;29(1):83–97.

35. Mudara ZJ, Mafini C. Linking strategy implementation to financial performance and firm survival in women- owned small to medium enterprises. Journal of Contemporary Management. 2022 Jan;19(1):168–97.

36. Ergo AE, O'Connor D, Mega TL. Status and determinants of poverty among women-owned micro-businesses in Wolaita zone, southern Ethiopia. Journal of Enterprising Communities. 2023 Nov 13;17(6):1439–66.

37. Deci EL, Ryan RM. Self-determination theory: A macrotheory of human motivation, development, and health. In: Canadian Psychology. 2008. p. 182–5.

38. Sheldon KM, Elliot AJ. Goal striving, need satisfaction, and longitudinal well-being: The self-concordance model. J Pers Soc Psychol. 1999;76(3):482–97.

39. Chemolli E, Gagné M. Evidence against the continuum structure underlying motivation measures derived from self-determination theory. Psychol Assess. 2014;26(2):575–85.

40. Ng JYY, Ntoumanis N, Thøgersen-Ntoumani C, Deci EL, Ryan RM, Duda JL, et al. Self-Determination Theory Applied to Health Contexts: A Meta-Analysis. Perspectives on Psychological Science. 2012 Jul;7(4):325–40.

41. Gagné M, Deci EL. Self-determination theory and work motivation. J Organ Behav. 2005 Jun;26(4):331–62.

42. Rahmadani VG, Schaufeli WB, Ivanova TY, Osin EN. Basic psychological need satisfaction mediates the relationship between engaging leadership and work engagement: A cross-national study. Hum Resour Dev Q. 2019 Dec 1;30(4):453–71.

43. Alfan M, Hendi Andriansyah Jurusan Pendidikan Ekonomi E, Ekonomika dan Bisnis F, Negeri Surabaya Jl Ketintang No U, Gayungan K, Sby K, et al. Prisma Sains: Jurnal Pengkajian Ilmu dan Pembelajaran Matematika dan IPA IKIP Mataram. 2022;10(3):598–608. Available from: https://doi.org/10.33394/j-ps.v10i3.5218

44. Piperopoulos P, Dimov D. Burst Bubbles or Build Steam? Entrepreneurship Education, Entrepreneurial Self-Efficacy, and Entrepreneurial Intentions. Journal of Small Business Management. 2015 Oct 1;53(4):970–85.

45. World Health Organization. Constitution of the World Health Organization. 1948.

46. Tennant R, Hiller L, Fishwick R, Platt S, Joseph S, Weich S, et al. The Warwick-Dinburgh mental well-being scale (WEMWBS): Development and UK validation. Health Qual Life Outcomes. 2007 Nov 27;5.

47. Diener E. Subjective well-being. Psychol Bull. 1984;95(3):542–75.

48. Ovharhe OH, Chukwuemeka SP. Sustainable Development Goals: Therapeutic Entrepreneurship and Mental Health Conditions. British Journal of Multidisciplinary and Advanced Studies. 2023 Feb 3;4(1):81–119.

49. Zappalà S, Swanzy EK, Toscano F. Workload and Mental Well-Being of Homeworkers: The Mediating Effects of Work-Family Conflict, Sleeping Problems, and Work Engagement. J Occup Environ Med. 2022 Oct 1;64(10):E647–55.

50. Bourdieu P. The forms of capital. In: Richardson，J., editor. Handbook of theory and research for the sociology of education. New York: NY: Greenwood Press; 1986. p. 241–58.

51. Coleman S, Carsky M. Women Owned Businesses and Bank Switching: The Role of Customer Service. The Journal of Entrepreneurial Finance. 1996 Dec 1;5(1):75–83.

52. Putnam R. The prosperous community: social capital and public life. Frontier issues in economic thought. 1997;3:211–2.

53. Guiso L, Sapienza P, Zingales L. NBER WORKING PAPER SERIES CIVIC CAPITAL AS THE MISSING LINK [Internet]. 2010. Available from: http://lists.repec.org/mailman/listinfo/nep-soc.

54. Grafton RQ, Knowles S. Social capital and national environmental performance: A cross-sectional analysis. Journal of Environment and Development. 2004 Dec;13(4):336–70.

55. Levkovski S and P V. Social capital and happiness at youth in social risk. 2022;3(24):25–41. Available from: www.ek-inst.ukim.edu.mk

56. Overcamp-Martini M. Theory for the Public Good? Social Capital Theory in Social Work Education. 2007.

57. Nahapiet J, Ghoshal S. Social Capital, Intellectual Capital, and the Organizational Advantage Author. Vol. 23, Source: The Academy of Management Review. 1998.

58. Mark S. Granovetter. The Strength of Weak Ties. American Journal of Sociology. 1973;78(6):1360–80.

59. Vamplew W. Sharing space: Inclusion, exclusion, and accommodation at the British golf club before 1914. J Sport Soc Issues. 2010;34(3):359–75.

60. Burke M, Marlow C, Lento T. Social network activity and social well-being. In: Proceedings of the SIGCHI Conference on Human Factors in Computing Systems. New York, NY, USA: ACM; 2010. p. 1909–12.

61. Kim D, Subramanian S V., Kawachi I. Bonding versus bridging social capital and their associations with self rated health: A multilevel analysis of 40 US communities. J Epidemiol Community Health (1978). 2006 Feb;60(2):116–22.

62. Collins CR, Neal JW, Neal ZP. Transforming Individual Civic Engagement into Community Collective Efficacy: The Role of Bonding Social Capital. Am J Community Psychol. 2014 Oct 30;54(3–4):328–36.

63. Simons M, Reijnders J, Janssens M, Lataster J, Jacobs N. Positive affect as mediator: The socioemotional selectivity theory applied to the association between bonding social capital and wellbeing in later life. J Soc Pers Relat. 2023 Jun 1;40(6):1854–74.

64. Rapeli M. Social capital in social work disaster preparedness plans: The case of Finland. Int Soc Work. 2018 Nov 1;61(6):1054–66.

65. Taormina RJ, Kin-Mei Lao S. Measuring Chinese entrepreneurial motivation:

Personality and environmental influences. International Journal of Entrepreneurial Behaviour & Research. 2007 Jun 19;13(4):200–21.

66. Su X, Liu S, Zhang S, Liu L. To be happy: A case study of entrepreneurial motivation and entrepreneurial process from the perspective of positive psychology. Sustainability (Switzerland). 2020 Jan 1;12(2).

67. Ryff CD. Entrepreneurship and eudaimonic well-being: Five venues for new science. J Bus Ventur. 2019 Jul 1;34(4):646–63.

68. Nikolaev B, Boudreaux CJ, Wood M. Entrepreneurship and Subjective Well-Being: The Mediating Role of Psychological Functioning. Entrepreneurship: Theory and Practice. 2020 May 1;44(3):557–86.

69. van der Vaart L, Van den Broeck A, Rothmann S, De Witte H. Motivational Profiles in Unemployment: A Self-Determination Perspective. Front Public Health. 2022 Apr 29;10.

70. Sikora J, Saha LJ. Vocational education, self-employment and burnout among Australian workers. Vol. 44, Australian Journal of Social Issues. 2009.

71. Johansson SI, Larsson D, Strandh M. The prevalence, characteristics and well-being of "necessity" self-employed and "latent" entrepreneurs: Findings from Sweden. International Journal of Entrepreneurship and Small Business. 2016;28(1):58–77.

72. Spencer LM, Spencer SM. COMPETENCE AT WORK Models for Superior Performance [Internet]. Available from: www.hippopublishing.com

73. Rindova V, Barry D, Ketchen DJ. Introduction to Special Topic Forum. Vol. 34, Academy of Management Review. Academy of Management; 2009. p. 477–91.

74. Teixeira PJ, Silva MN, Mata J, Palmeira AL, Markland D. Motivation, self-determination, and long-term weight control. Vol. 9, International Journal of Behavioral Nutrition and Physical Activity. 2012.

75. Hood C, Patton R. Exploring the role of psychological need fulfilment on stress, job satisfaction and turnover intention in support staff working in inpatient mental health hospitals in the NHS: a self-determination theory perspective. Journal of Mental Health. 2022;31(5):692–8.

76. Carsrud A, Brännback M. Entrepreneurial Motivations: What Do We Still Need to Know? Journal of Small Business Management. 2011 Jan;49(1):9–26.

77. Zhu Y, Collins A, Xu Z, Sardana D, Cavusgil ST. Achieving aging well through senior entrepreneurship: a three-country empirical study. Small Business Economics. 2022 Aug 1;59(2):665–89.

78. Al-Jubari I, Hassan A, Liñán F. Entrepreneurial intention among University students in Malaysia: integrating self-determination theory and the theory of planned behavior. International Entrepreneurship and Management Journal. 2019 Dec 1;15(4):1323–42.

79. Ryan RM, Deci EL. A self-determination theory approach to psychotherapy: The motivational basis for effective change. In: Canadian Psychology. 2008. p. 186–93.

80. Rigby CS, Ryan RM. Self-Determination Theory in Human Resource Development: New Directions and Practical Considerations. Adv Dev Hum Resour. 2018 May 20;20(2):133–47.

81. Morse KF, Fine PA, Friedlander KJ. Creativity and Leisure During COVID-19: Examining the Relationship Between Leisure Activities, Motivations, and Psychological Well-Being. Front Psychol. 2021 Jul 5;12.

82. Nielsen K, Nielsen MB, Ogbonnaya C, Känsälä M, Saari E, Isaksson K. Workplace resources to improve both employee well-being and performance: A systematic review and meta-analysis. Vol. 31, Work and Stress. Routledge; 2017. p. 101–20.

83. Robertson I, Cooper C. Well-Being and Employee Engagement. In: Well-Being. London: Palgrave Macmillan UK; 2011. p. 27–37.

84. Jansson I, Gunnarsson AB. Employers' views of the impact of mental health problems on the ability to work. Work. 2018;59(4):585–98.

85. Kotera Y, Conway E, Van Gordon W. Mental health of UK university business students: Relationship with shame, motivation and self-compassion. Journal of Education for Business. 2019 Jan 2;94(1):11–20.

86. Hessels J, Rietveld CA, Roy Thurik A, Van Der Zwan P. Depression and entrepreneurial exit. In: Academy of Management Learning and Education. George Washington University; 2018. p. 323–39.

87. Simmons P, Dalton H. Influences of rurality on small business owner mental well-being in regional NSW. Australian Journal of Rural Health. 2023 Apr 1;31(2):171–81.

88. Aftan Y, Hanapi M. The Impact of Entrepreneurial Motivation on Small Business Performance in Iraq. International Journal of Academic Research in Business and Social Sciences. 2018 Feb 18;8(1).

89. Bell M, Thach L, Fang F. Examining motivations and challenges of black wine entrepreneurs using the push–pull theory of entrepreneurship. International Journal of Wine Business Research. 2023 Jul 17;35(3):445–66.

90. Kirkwood J. Motivational factors in a push-pull theory of entrepreneurship. Gend

Manag. 2009;24(5):346–64.

91. Ambarwati T, Tri DJATMIKA E, Handayati P. ENTREPRENEURIAL VALUES ON BUSINESS PERFORMANCE: ENTREPRENEURIAL COMMITMENT AS A MEDIATION. International Journal of Environmental, Sustainability, and Social Sciences [Internet]. 2022;3(2):234–41. Available from: https://journalkeberlanjutan.com/index.php/ijesss

92. Ulfa Sentosa S. Analyzing The Entrepreneur Process of Motivation, Resource Allocation, and Institutional for Poor Households in Rural West Sumatera. 2018.

93. Arslan G, Yıldırım M. A Longitudinal Examination of the Association Between Meaning in Life, Resilience, and Mental Well-Being in Times of Coronavirus Pandemic. Front Psychol. 2021 Apr 28;12.

94. Conduah AK, Essiaw (PhD) MN. Resilience and entrepreneurship: a systematic review. F1000Res. 2022 Mar 23;11:348.

95. Bondeli J V., Havenvid MI, Solli-Sæther H. Towards understanding the role of social capital for value creation in business relationships: The case of Russia. In: Bidrag innen kundeverdi og marked. Universitetsforlaget; 2021. p. 194–216.

96. Roy A. Interpersonal Emotion Regulation and Emotional Intelligence: A Review. International Journal of Research Publication and Reviews. 2023;04(02):623–7.

97. Kelloway EK. Mental health in the workplace: Towards evidence-based practice. In: Canadian Psychology. Canadian Psychological Association; 2017. p. 1–6.

98. Gao J, Weaver SR, Dai J, Jia Y, Liu X, Jin K, et al. Workplace social capital and mental health among chinese employees: A multi-level, cross-sectional study. PLoS One. 2014 Jan 3;9(1).

99. Patulny R V., Lind Haase Svendsen G. Exploring the social capital grid: Bonding, bridging, qualitative, quantitative. Vol. 27, International Journal of Sociology and Social Policy. 2007. p. 32–51.

100. Agger A, Jensen JO. Area-based Initiatives—And Their Work in Bonding, Bridging and Linking Social Capital. European Planning Studies. 2015 Oct 3;23(10):2045–61.

101. Cao Q, Lu Y, Dong D, Tang Z, Li Y. The roles of bridging and bonding in social media communities. Journal of the American Society for Information Science and Technology. 2013 Aug;64(8):1671–81.

102. Yezza H, Chabaud D, Dana LP, Maalaoui A. The impact of bridging social capital in family firms' performance: exploring the mediation role of successor's social skills.

International Journal of Entrepreneurial Behavior & Research. 2021 Nov 2;27(8):2009–27.

103. Pihl P, Albertsen K, Hogh A, Andersen LPS. Social capital and workplace bullying. Work. 2017;57(4):535–45.

104. Crescenzi R. The Bright Side of Social Capital: How “Bridging” Makes Italian Provinces More Innovative. 2011.

105. Kishimoto Y, Suzuki E, Iwase T, Doi H, Takao S. Group involvement and self-rated health among the Japanese elderly: an examination of bonding and bridging social capital. BMC Public Health [Internet]. 2013;13(1):1189. Available from: http://www.biomedcentral.com/1471-2458/13/1189

106. Usher Mitchell C, LaGory M. Social Capital and Mental Distress in an Impoverished Community. City Community. 2002;1(2):199–222.

107. Gong S, Xu P, Wang S. Social capital and psychological well-being of Chinese immigrants in Japan. Int J Environ Res Public Health. 2021 Jan 2;18(2):1–12.

108. Keyes CLM. The mental health continuum: From languishing to flourishing in life. J Health Soc Behav. 2002;43(2):207–22.

109. Tan JJ, Litschert RJ. Environment-Strategy Relationship and Its Performance Implications: An Empirical Study of the Chinese Electronics Industry. Vol. 15, Source: Strategic Management Journal. 1994.

110. Gódány Z, Machová R, Mura L, Zsigmond T. Entrepreneurship Motivation in the 21st Century in Terms of Pull and Push Factors. TEM Journal. 2021 Feb 1;10(1):334–42.

111. Vuković K, Kedmenec I, Postolov K, Jovanovski K, Korent D. THE ROLE OF BONDING AND BRIDGING COGNITIVE SOCIAL CAPITAL IN SHAPING ENTREPRENEURIAL INTENTION IN TRANSITION ECONOMIES. Vol. 22, Management. 2017.

112. Hairudinor H, Daradjat AA, Asman N. The impact of entrepreneurial education and competitiveness. Cypriot Journal of Educational Sciences. 2020;6(15):1732–43.

113. Cook RG, Belliveau P. The influence of human capital attributes in microenterprise training. Journal of Small Business and Enterprise Development. 2004 Dec 1;11(4):467–73.

114. Ma H, Zhang YC, Butler A, Guo P, Bozward D. Entrepreneurial performance of new-generation rural migrant entrepreneurs in China. International Journal of Entrepreneurial Behavior & Research. 2022 Mar 1;28(2):412–40.

115. Maireva C, Rejoyce Magomana N. The Influence of Endogenous Factors on Entrepreneurial Success Among Youths in Masvingo Urban, Zimbabwe. EAST AFRICAN JOURNAL OF EDUCATION AND SOCIAL SCIENCES. 2021 May 24;(Issue 2 (April to June 2021)):179–89.

116. Coste J, Quinquis L, Audureau E, Pouchot J. Non response, incomplete and inconsistent responses to self-administered health-related quality of life measures in the general population: Patterns, determinants and impact on the validity of estimates - a population-based study in France using the MOS SF-36. Health Qual Life Outcomes. 2013 Mar 13;11(1).

117. Felix R. The impact of scale width on responses for multi-item, self-report measures. Journal of Targeting, Measurement and Analysis for Marketing. 2011 Sep;19(3–4):153–64.

118. R. Y. Cavana, Brian L. Delahaye, Uma Sekaran. Applied Business Research: Qualitative and Quantitative Methods. John Wiley & Sons; 2001.

119. Hayes AF. Introduction to mediation, moderation, and conditional process analysis : a regression-based approach. 507 p.

120. Hessels J, van Gelderen M, Thurik R, Hessels J, Thurik R, van Gelderen M. Entrepreneurial aspirations, motivations, and their drivers Entrepreneurial aspirations, motivations, and their drivers Keywords Entrepreneurial motivations • Entrepreneurial aspirations • Global Entrepreneurship Monitor JEL Classifications H50 • L21 • L26 • Ml 3. Vol. 17, Development and Institutions. 2008.

Figure 1

Click here to access/download;Figure;Fig_1_Theoretical_Model.tif

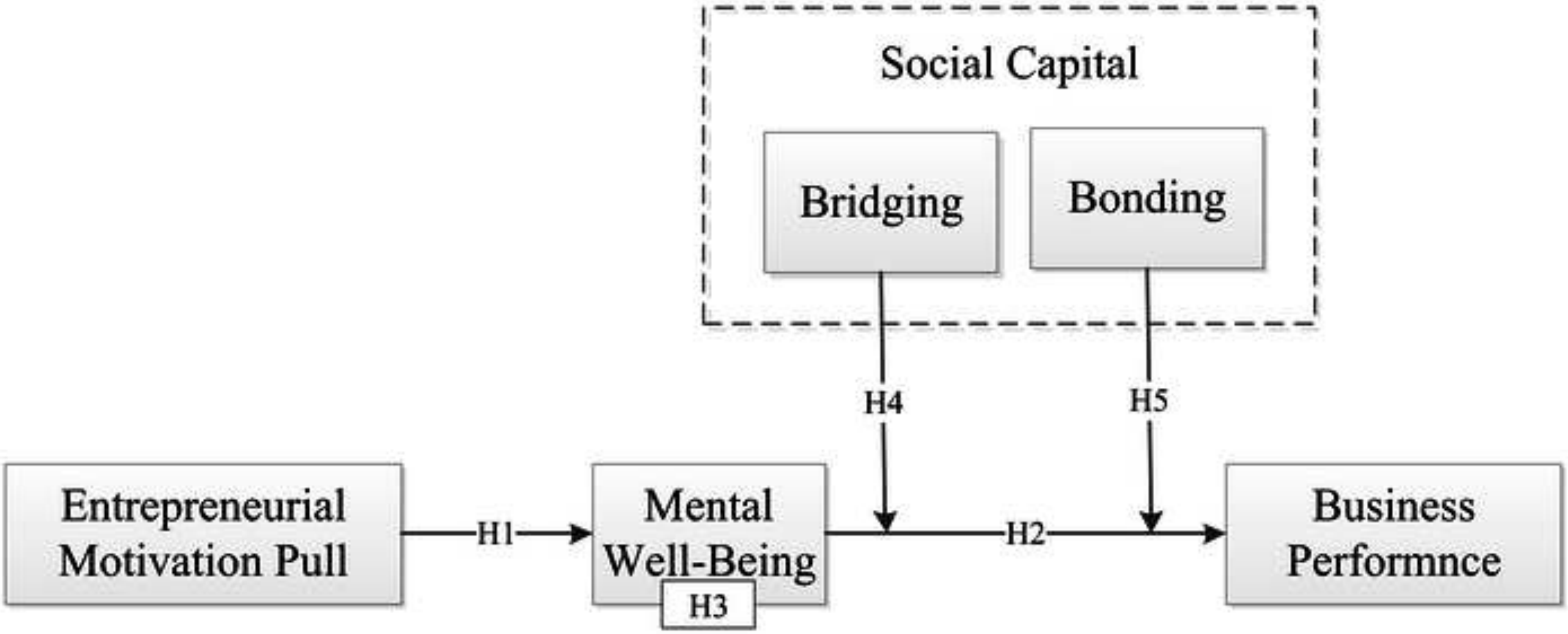

Figure 2

Click here to access/download;Figure;Fig_2_Mediated_Moderation_Model (Model 16).TIF

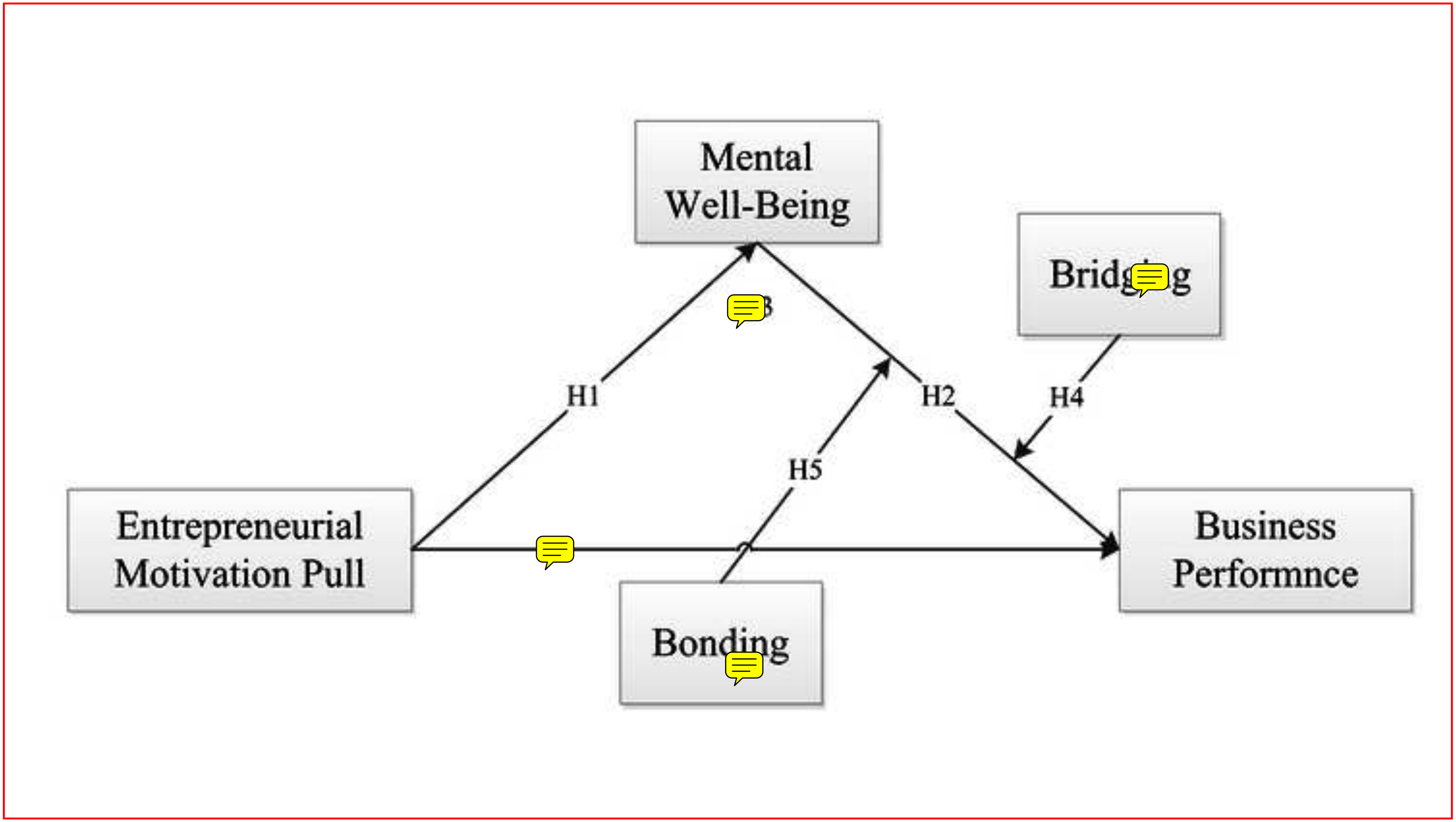

Figure 3

Click here to access/download;Figure;Fig_3_Moderating_effects_of_Social_Capital.tif

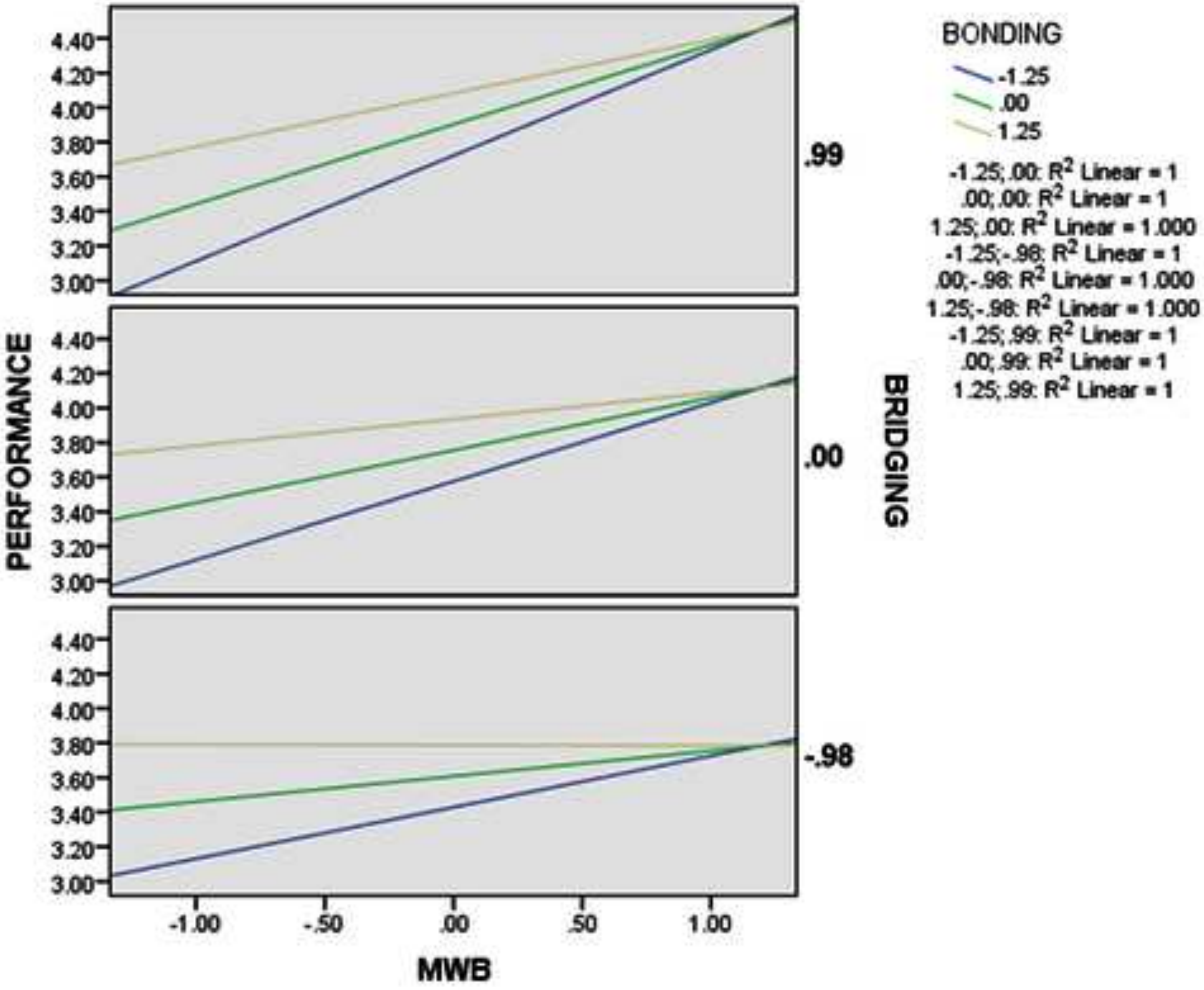

Supporting files list

Click here to access/download
**Supporting Information**
support files list.docx

data

Click here to access/download
**Supporting Information**
S1_Dataset.xls
