# Supplementary material for: Pull motivation and well-being as drivers of entrepreneurial success: The moderating role of social capital
Source: PLoS One. 2025 Aug 4;20(8):e0327894. doi: 10.1371/journal.pone.0327894 (PMC12321112; doi:10.1371/journal.pone.0327894)
Supplement: Attachment — Submitted filename: response to reviewers.docx [file pone.0327894.s005.docx]

August 11, 2024

Dear Dr. María José Ibáñez,

Below you will find our response and rebuttal to each point raised by the academic editor and reviewers.

Response to Academic Editor

1. *Please ensure that your manuscript meets PLOS ONE's style requirements, including those for file naming.*

Thank you for the feedback. In response to this request, we have made a lot of changes to our manuscript. For example, we have reformatted the title of the manuscript, ensuring that it is written in sentence style.

2. *Please provide additional details regarding participant consent. In the ethics statement in the Methods and online submission information.*

Thank you for your feedback. As data collection was exempted by the IRB of SolBridge, participant consent was waived. This is noted on lines 433-445.

3. *When completing the data availability statement of the submission form, you indicated that you will make your data available on acceptance.* *We strongly recommend all authors decide on a data sharing plan before acceptance, as the process can be lengthy and hold up publication timelines.*

We will decide on a data sharing plan based on your suggestion before accepting it and declare it in the new cover letter.

4. *Please include captions for your Supporting Information files at the end of your manuscript, and update any in-text citations to match accordingly.*

We added the captions for the dataset and appendix at the end of the manuscript, which we named “**S1 Appendix. Female Entrepreneurial Well-Being Questionnaire.**" and “**S1 Dataset. Full dataset.**" An in-text citation was provided at line 1205-1206.

Response to Reviewer 1

1. *Keywords are missing.*

Thank you for your comment! We apologize for the oversight. We have added the keywords below the abstract.

2. *The introduction is not as extensively written as it should have been and it should be brimmed with more citations and history.*

According to your suggestion, We have rewritten the introduction and included more citations and history.

3. *Variables should have been discussed deeply i.e. Female Entrepreneurial Motivation Pull, Business Performance in Women-owned Businesses, Self-Determination Theory, Mental Health and Well-Being (MWB), Social Capital.*

We have rewritten the literature review.

4. *Methodology:*
   a. *In Table 5 some variables are insignificant as per p-values, however, all hypotheses have been shown supported under the regression analysis heading.*

In the results of Table 5, the constant term does not significantly affect the results for the dependent variable PERFORMANCE and does not affect the results. The effect of the independent variable PULL on the dependent variable PERFORMANCE is not significant, reflecting the fact that MWB fully mediates the relationship between PULL and PERFORMANCE. Whereas the later determination of significance is based on the determination at the 0.05 level of significance, the significant effect of INTERACTION1 and INTERACTION2 on PERFORMANCE at the 0.05 level of significance reflects the fact that both BRIDGING and BONDING have a significant moderating effect on the effects of MWB and PERFORMANCE. Thus, the subsequent hypotheses are valid, specifically H4, H5 are supported.

5. *Overall Comments:*

*The paper is outclassed; however, the methodology has not been given whether it was cross-sectional, longitudinal, qualitative, or quantitative research type. When was the research work completed?*

In the section of the text describing the data collection, we would clearly state that "the questionnaire was collected over a period of two months. The questionnaire was used to obtain cross-sectional data and then analyzed quantitatively".(line 446-449)

*As per the model “bridging and bonding” are the moderators and “wellbeing” is a mediator and the model has serial moderation and discussion. In the second model, the arrow reflects wrong, however when they moderate the variables then it should also moderate the IV as well, however, they indicate only “Mediation Effects”.*

*Hypothesis 3 is wrong; it should have been generated between IV and IV.*

We just strictly used the Model No 14 in the SPSS PROCESS macro.

Response to Reviewer 2

1. *Provide a last paragraph in the introduction to describe the structure of the manuscript;*

Thank you for your suggestion! We have added the paragraph at the end of introduction.(line 105-115)

2. *Lack of Theoretical foundation, please add this section, Whisthery will suuport your conceptual model?*

We take the Conservation of Resources Theory as our theoretical foundation.

3. *The literature review should be more elaborated in order to link the main issues mentioned.*

Thank you for your kind comments! We rewrite the literature and add some paragraphs to illustrate the relevance of the previous literature review to this study, as well as the similarities and differences between existing research and this study. *Which kinds of procedures were adopted to reach this greatmark, especially data collection?*

The process of data collection used a convenience sampling, which we will also refine into the paper.

4. *Please show your measurement model and structrual model.*

Since we are using SPSS PROCESS MACRO, directly importing the observed variables into the existing model will directly generate the relevant data of the model and variables, so there is no structural model.

5. *Provide implications as well as contributions of this manuscript for the reader.It is necessary to emphasize andwrite more clearly the managerial contributions. Also,essential to add theoretical implications in this regard.*

Based on the suggestions, we improved the content of the contributions and implications.

6. *Add a research instrument with appropriate sources(questionnaires) before the details reference section.*

As suggested, we attached the questionnaire in the appendix before the reference.

7. *Minor issues relate to the structure and readability of the study*

We have rechecked and corrected the grammatical errors.

Response to Reviewer 3

1. *Abstract*

*The abstract does not include all key aspects such as significance, contribution, and limitations of the study.*

We refined the abstract.

2. *Introduction*

*Several statements are not supported with references such as the first sentence.*

*The language quality of the manuscript needs to be improved. You may need to seek assistance from a professional editing service to improve clarity, readability, and overall flow. An example of a grammatical error (reference) is on page 9 “Although previous studies have explored the relationship between different types of entrepreneurial motivation and different types of business performance [6]. However, it did not delve deeper into the specific mechanisms by which entrepreneurial motivation pull affects business performance.”*

*The content and organization of the introduction do not align with scholarly publications. For example, the gap in existing literature that your study aims to address is not clearly indicated or developed. As an example, the article should have emphasized the lack of comprehensive research regarding the role of social capital in shaping the relationship between mental health, well-being, and business performance among female Chinese entrepreneurs. This idea could be developed and supported with adequate references. Further and more critical information is missing.*

We rewrote the introduction.

3. *Literature Review*

*Concepts in the review are situated in ‘developing countries’ and are not oriented to support or describe the specific work context of Chinese female entrepreneurs. There is no attention to developing background information that is specific to the study. For example, the author mentions that “Research has shown that resource restrictions, business model pivots, family embeddedness, and demographic variables all have a major influence on the performance of women-owned enterprises”. How do these variables relate to the Chinese context, what evidence and research supports such claims in the context from which data is drawn in your study, and how does a reader foreign to your context understand the socio-economic implications of these variables? These are examples of some questions concerning the background of the study that may need further attention.*

We explain this in the introduction as well as the literature review: the reasons why Chinese entrepreneurs were studied.

*Unfortunately, the literature review consists of relevant but disconnected theoretical concepts and ideas and does not synthesize existing knowledge to establish the context within which your study is situated. This contextualization is essential for readers to understand the significance of your research and its contribution to the field. It is not building an argument towards a research gap nor indicating the position of your research within the broader scholarly conversation and hence does not demonstrate your understanding of the relevant literature. One of the primary functions of a literature review is to identify gaps, contradictions, or limitations in the existing literature. By critically analyzing previous research, you can pinpoint areas where existing findings are inconsistent or inconclusive and hence your investigation is needed to fill the gap.*

We reorganized the literature review.

*The Self-Determination Theory is indicated as “an important theoretical foundation for entrepreneurial studies”; however, there is no explanation of how it is used as the theoretical framework for your study. For example, will it be used to guide the formulation of hypotheses that specify the expected relationships between variables based on theoretical principles? By grounding your hypotheses in established theory, you provide a theoretical rationale for your research questions and predictions. Alternatively, SD theory informs the choice of statistical techniques and methods for data analysis. By selecting appropriate analytical methods that are consistent with the theoretical framework, you ensure that your findings are interpreted in light of theoretical expectations and contribute to theoretical advancement. In other words, you need to ensure that your quantitative study is theoretically grounded and that SD theory guides all aspects of the research process from conceptualization to interpretation of findings. Grounding your study in established theory enhances the generalizability and external validity of your findings. By building on a strong theoretical framework, you provide a basis for comparing your results with previous research and extending conclusions to broader populations or contexts.*

We take the Conservation of Resources Theory which better explain the complex relationship between the various variables in this study as our theoretical foundation instead of SDT.

*On page 21, you mention that social capital plays a moderating role and “this mechanism is supported by several studies that have explored it from various perspectives.” without supporting this statement with adequate references. The support of the research hypotheses is not well organized to convey an argument that leads to the need to investigate the hypothesis. Instead, this section consists of relevant but disconnected ideas.*

We’d like to add a paragraph after the sentence “this mechanism is supported by several studies that have explored it from various perspectives” to elaborate on the research that supports this statement.

*Suggesting a model that is not based on the literature or the findings of the study is not appropriate.*

We used conservation of resources theory to fully support the complex relationships among the variables in our theoretical model.

4. *Methodology*

*While your method claims to use random sampling, the reliance on an online survey platform may introduce sampling bias. Not all individuals have equal access to or use of online platforms, which could result in underrepresentation or exclusion of certain segments of the population, such as those with limited internet access or technology literacy. This can undermine the representativeness of the sample and limit the generalizability of the findings. Respondents who choose to participate in online surveys may differ systematically from those who do not, leading to self-selection bias. For example, only females who have issues or strong opinions on the subject may be more likely to respond, skewing the sample towards certain characteristics or attitudes. This can compromise the external validity of the findings. The quality and reliability of responses collected through an online survey platform may vary. Filtering respondents based on IP addresses in mainland China may limit the diversity of the sample and overlook Chinese female entrepreneurs residing outside of China who with cross-border business interests, potentially narrowing the scope of the study and overlooking valuable perspectives. Otherwise, it is important to justify the limitations of the study and indicate them upfront. The description of the data collection method should provide sufficient detail about the measures taken to control for potential biases or ensure the quality of the data collected. Without adequate control measures, there is a heightened risk of bias, errors, or inconsistencies in the data, compromising the reliability and validity of the study results.*

Firstly, Chinese women entrepreneurs living abroad are not included in the study because the variables in the study are limited by socio-economic and cultural factors.

The specific impact of socio-economic and cultural factors was added to the article.

It is also acknowledged in the conclusion that the generalizability of the results of the article may be limited due to the specificity of the sample caused by the particular socio-cultural and economic factors.

*You mention that “After careful review and elimination of unreasonable answers” without explaining what is considered to be ‘unreasonable’ or detailing your exclusion criteria.*

The data specifically screened was done in such a way that questionnaires with a large number of repetitive options as well as a large number of regular repetitive options were mainly eliminated, as the rigor of the answers given in these questionnaires was questioned. These were also added to the article

*On page 28, you mention that ‘we controlled for participants’ education level and entrepreneurial experience in the following data analysis’ without explaining how this is done.*

We use them as control variables

5. *Descriptive statistics*

*Although this section has better empirical content than previous ones, it draws attention to a lack of support for all six hypotheses in the literature.*

We adopted the conservation of resources theory as a theoretical basis to connect all the variables.

6. *Findings and Discussion*

*Overall, the discussion section does not build adequate evidence drawn from the findings in connection with the literature. I do not see a convincing argument or organization per hypothesis.*

*The statement - “entrepreneurial motivation positively affects MWB. Consequently, enhanced MWB significantly boosts business performance. MWB fully mediates the relationship between entrepreneurial motivation pull and business performance” - implies causal relationships between entrepreneurial motivation, mental well-being (MWB), social capital, and business performance. However, without longitudinal or experimental data, it's challenging to establish causality definitively. The relationships described may be bidirectional or influenced by other factors not accounted for in the analysis; however, this is not indicated or justified.*

We further strengthened the theoretical support for the hypothesized relationships by introducing resource conservation theory and rewriting the literature review section.

*On page 38, you emphasize the need for ‘addressing entrepreneurial mental health as a strategy” but do not explain how or support this with examples and references.*

We add a paragraph according to your suggestion in the contribution and implications part.

7. *Conclusion*

*The conclusion is too short and does not cover the requirements of an academic article.*

*There is no mention of the theoretical contribution and practical implications.*

We rewrote the conclusion section, especially adding theoretical contributions and practical applications.

*Finally, attending to the above concerns (I have not covered all issues due to time restrictions) would help in refining the manuscript and strengthening its contribution to the academic discourse.*

In sum, we express our gratitude to you and all the reviewers for the valuable feedback, which we firmly believe has significantly enhanced the quality of our manuscript.

Sincerely,

Shibo Li

Corresponding Author
