# Supplementary material for: Pull motivation and well-being as drivers of entrepreneurial success: The moderating role of social capital
Source: PLoS One. 2025 Aug 4;20(8):e0327894. doi: 10.1371/journal.pone.0327894 (PMC12321112; doi:10.1371/journal.pone.0327894)
Supplement: Attachment — Submitted filename: response_to_reviewers_auresp_2.docx [file pone.0327894.s006.docx]

December 9, 2024

Dear Dr. María José Ibáñez,

Below is our response and rebuttal to the significant areas of revision raised by the academic editor and reviewers during the second round of modifications

*1. Title and Abstract: The title needs to be more concise. Additionally, the abstract should be expanded to include the study's significance, contribution, and limitations, which are currently missing.*

We have made the title more concise. At the same time, the abstract section has been expanded to include the significance, contribution, and limitations of the study, specifically see lines 29-43.

*2. Introduction and Literature Review: The introduction lacks sufficient references to support its statements and needs to clearly identify the research gap your study addresses.*

We have revised the introduction section, adding more references to support our statements. Additionally, we have specifically identified the research gaps that our study addresses, which are mainly reflected in three key areas. This is noted on lines 48-132.

*The literature review is criticized for not adequately situating the study within the specific context of Chinese female entrepreneurs. It should be more coherent, linking concepts to the study’s objectives and developing a stronger argument towards the research gap. Additionally, the theoretical foundation, particularly the application of Self-Determination Theory (SDT), needs to be clarified and integrated into the study's design, hypothesis formulation, and data analysis.*

We have thoroughly revised the literature review section and integrated it with the research hypotheses. We have changed the main theory from SDT to COR and Social Capital Theories. In the review, we have placed our study firmly within the specific context of Chinese female entrepreneurs and woven the various elements together using Conservation of Resources Theory (COR) to create a cohesive narrative. For specific content, please refer to lines 133 to 375.

*3. Methodology: The methodology section needs to address potential biases introduced by the use of an online survey platform and provide more detail on the data collection process, including criteria for excluding responses and controlling variables like education level and entrepreneurial experience. The study's design, particularly the sampling method and its implications for the representativeness of the results, should be justified.*

We have refined the methodology section and provided more details about the online survey platform and the data collection process. For specific content, please refer to lines 379 to 436.

*4. Results and Discussion: The discussion section requires a more thorough integration of the findings with existing literature and a clearer organization around the study's hypotheses. The current interpretation of the results implies causality without adequate justification, and this needs to be addressed by either adjusting the claims or providing a stronger methodological basis for causal inference.*

In the discussion section, we have thoroughly integrated the research findings with existing literature, thereby strengthening the causal inference. For specific details, please refer to lines 662-696.

*5. Conclusion: The conclusion is too brief and should be expanded to include a discussion of the study's theoretical contributions, practical implications, and the significance of the findings.*

We have expanded the conclusion section, discussing the contributions and significance of the study from theoretical, practical, and policy perspectives. For specific content, please refer to lines 697-721.

*6. Overall Structure and Clarity: The manuscript requires improvements in language quality, organization, and the inclusion of key elements such as a more detailed introduction, theoretical foundation, and a clearer presentation of the research model. Minor revisions related to readability, structure, and the inclusion of supplementary materials like the research instrument are also necessary.*

We have made comprehensive improvements in terms of language quality and organizational structure, refining the sections on introduction, theory and hypotheses, methodology, results and discussion, as well as conclusion to enhance the readability and clarity of the article.

In sum, we express our gratitude to you and all the reviewers for the valuable feedback, which we firmly believe has significantly enhanced the quality of our manuscript.

Sincerely,

Shibo Li

Corresponding Author
